# Costs of position, velocity, and force requirements in optimal control induce triphasic muscle activation during reaching movement

**DOI:** 10.1038/s41598-021-96084-2

**Published:** 2021-08-19

**Authors:** Yuki Ueyama

**Affiliations:** Department of Mechanical Engineering, National Defense Academy of Japan, Yokosuka, Kanagawa Japan

**Keywords:** Dynamical systems, Neurophysiology, Musculoskeletal models

## Abstract

The nervous system activates a pair of agonist and antagonist muscles to determine the muscle activation pattern for a desired movement. Although there is a problem with redundancy, it is solved immediately, and movements are generated with characteristic muscle activation patterns in which antagonistic muscle pairs show alternate bursts with a triphasic shape. To investigate the requirements for deriving this pattern, this study simulated arm movement numerically by adopting a musculoskeletal arm model and an optimal control. The simulation reproduced the triphasic electromyogram (EMG) pattern observed in a reaching movement using a cost function that considered three terms: end-point position, velocity, and force required; the function minimised neural input. The first, second, and third bursts of muscle activity were generated by the cost terms of position, velocity, and force, respectively. Thus, we concluded that the costs of position, velocity, and force requirements in optimal control can induce triphasic EMG patterns. Therefore, we suggest that the nervous system may control the body by using an optimal control mechanism that adopts the costs of position, velocity, and force required; these costs serve to initiate, decelerate, and stabilise movement, respectively.

## Introduction

**Figure 1:**
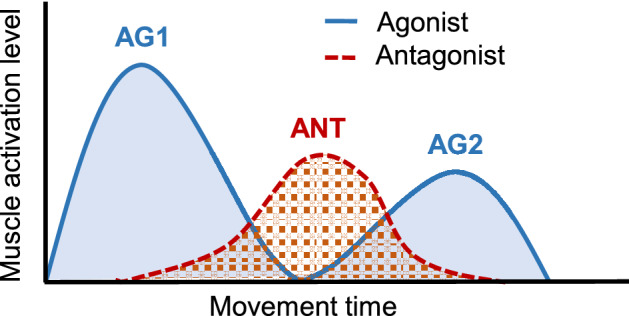
Triphasic muscle activation pattern during reaching movement. AG1, AG2, and ANT indicate the sequence of bursts for the pair of agonist (AG1 and AG2) and antagonist (ANT) muscles.

Mammalian biomechanical motor control comprises multiple joints and muscles that form redundant systems with multiple degrees of freedom. To move, the nervous system must overcome the problem of redundancy and determine a movement trajectory and muscle activation pattern (i.e. the electromyogram [EMG] pattern) that involves pairs of agonist and antagonist muscles. In both single-joint and multi-joint reaching movements, the agonist and antagonist muscles typically burst in triphasic patterns in an alternating manner^1,2^. Thus, the muscles are presumably tuned selectively according to movement direction, similar to isometric force production^3^. Agonist and antagonist muscles are activated in a triphasic alternating pattern (Fig. 1). Agonist muscles are strongly activated at movement onset (Fig. 1, AG1); the antagonist muscles then show single peaks at the midpoint of movement (Fig. 1, ANT), following delayed peaks in velocity profiles^4^. Subsequently, the agonist muscles are weakly reactivated (Fig. 1, AG2). The AG1 and ANT burst pair determines the increase and decrease in acceleration, respectively; the AG2 burst increases deceleration^5^ or dampens oscillations that occur at the end of movement^6^. Thus, the triphasic EMG pattern is not directly related to movement amplitude, speed, or duration; it is directly related to acceleration and deceleration^7^. This simple relationship between the EMG pattern and movement implies that the nervous system can easily determine the muscle activation patterns needed to produce specific movements^8^. Indeed, single-neuron and population-level activities in the primary motor cortex (M1) show triphasic changes in temporal pattern and instantaneous directionality, similar to the EMG^9,10^; however, the function of these neurons is currently controversial, with research suggesting that they determine the speed and direction of hand motion^11^, acceleration^12^, or joint movement and muscle force^13^. The triphasic EMG pattern is induced by the nervous system as an open-loop control, although the central program is currently unknown.

In the computational motor control domain, optimal feedback control (OFC) theory has been proposed^14–16^, which predicts various movement phenomena (e.g. obstacle avoidance^17^, adaptation to novel tasks^18^, stiffness modulation^19^, specific muscle actions^20,21^, and the manipulation of complex objects^22^); it also predicts neural representation in M1^23^. The neural activity in M1 is presumably optimised for the musculoskeletal structure using an OFC-like cost function^24–26^. Most trajectory planning models (e.g. minimum-jerk^27^ and minimum-torque-change^28^ models) are presumably unable to predict muscle actions^29^, while a simple model of optimal control can predict EMG patterns in step-tracking wrist movement^30^. Although the minimum acceleration with constraints model can predict triphasic muscle activity^31^, it predicts only the durations of muscle activation and inactivation; it cannot explain how redundant muscles are recruited to specific motor tasks, or how they modulate the bursts of each muscle. Thus far, various studies have evaluated several cost functions in optimal control to generate a point-to-manifold reaching task that leaves the target underdetermined^32–34^. They suggested that the reaching trajectories are generated by the optimality principle with compositions of multiple costs (e.g. kinematic smoothness and mechanical energy consumption). However, the cost function that can predict the EMG patterns in arm movement remains unknown.

In this study, we re-examined the triphasic muscle activation patterns observed in EMGs during arm movement from the perspective of optimal control using an OFC-like cost function that consisted of terminal requirement costs with minimised neural input to predict the muscle activation patterns. Then, we performed numerical simulations involving application of an iterative linear–quadratic–Gaussian (ILQG) method^35^, which approximates OFC, to physiological arm dynamics with a realistic muscle model for macaque monkeys. This revealed that the optimal control could selectively tune muscles according to movement direction, and it indicated an interlaced cost based on combinations of the terminal requirement of the end-point position, velocity, and force under minimisation of neural input during movement. The control induced triphasic muscle activation patterns similar to the patterns in recorded EMGs, only under certain conditions validating the position, velocity, and force costs. Thus, each cost corresponded to each burst of the agonist and antagonist muscles (i.e. AG1, ANT, and AG2). Consequently, we suggest that the neural system controls the body by using an optimal control mechanism based on a cost function that consists of position, velocity, and force requirements. These requirements correspond with the first (AG1), second (ANT), and third (AG2) muscle activation bursts, which serve to initiate, decelerate, and stabilise movement, respectively.

## Results

We simulated movement using an approximate OFC^35^ and a two-joint six-muscle arm model^19,36,37^ (i.e. shoulder flexor [SF], shoulder extensor [SX], elbow flexor [EF], elbow extensor [EX], biarticular flexor [BF], and biarticular extensor [BX] muscles). The simulation reproduced a centre-out reaching task, which required moving the hand to targets aligned 8 cm from the initial hand position. For base setting, we fixed the simulation duration at 500 ms and fixed the movement duration at 400 ms to fit the experimental data^2,19^. Then, we assumed four situations involving the various cost weights in Eq. (10) as Cases 1–4. In Case 1, the positional cost was active, and movement was only constrained in the terminal position. In Case 2, the cost terms of the position and velocity were active. This required movement under kinematic constraints. In Case 3, the force actively inhibited the end-point force at the target position. Finally, in Case 4, cost terms of the position, velocity, and force were active, which required regulation of the end-point position, velocity, and force states at the movement end. The task required stopping at the target without force generation.

**Figure 2:**
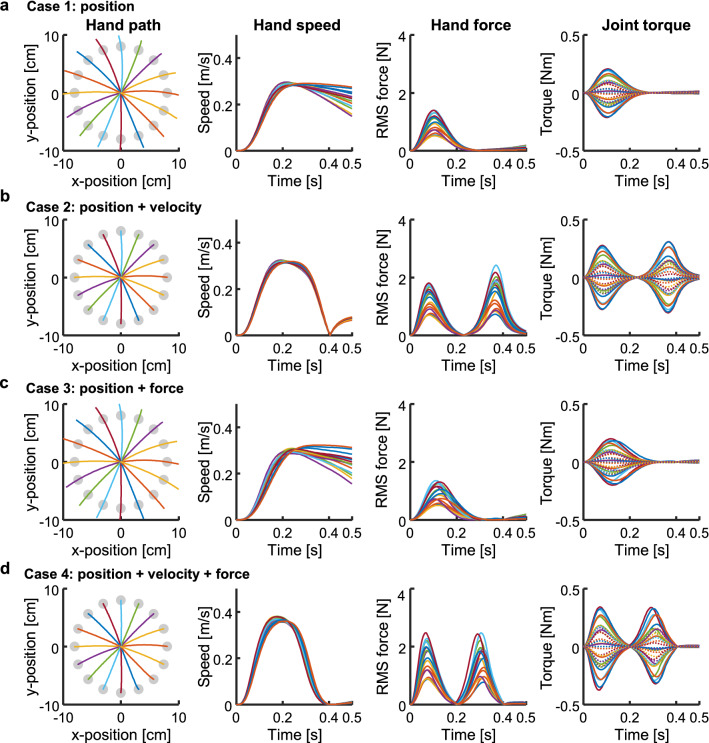
Centre-out movement for 16 targets. Columns from left to right are the hand paths, hand speed profiles, root mean squared (RMS) hand force profiles, and joint torque profiles. In the hand paths, target positions are shown as filled circles. In the joint torque profiles, solid and dotted lines indicate torques of the shoulder and elbow joints, respectively. Cases 1–4 are in rows (**a**)–(**d**), respectively.

**Figure 3:**
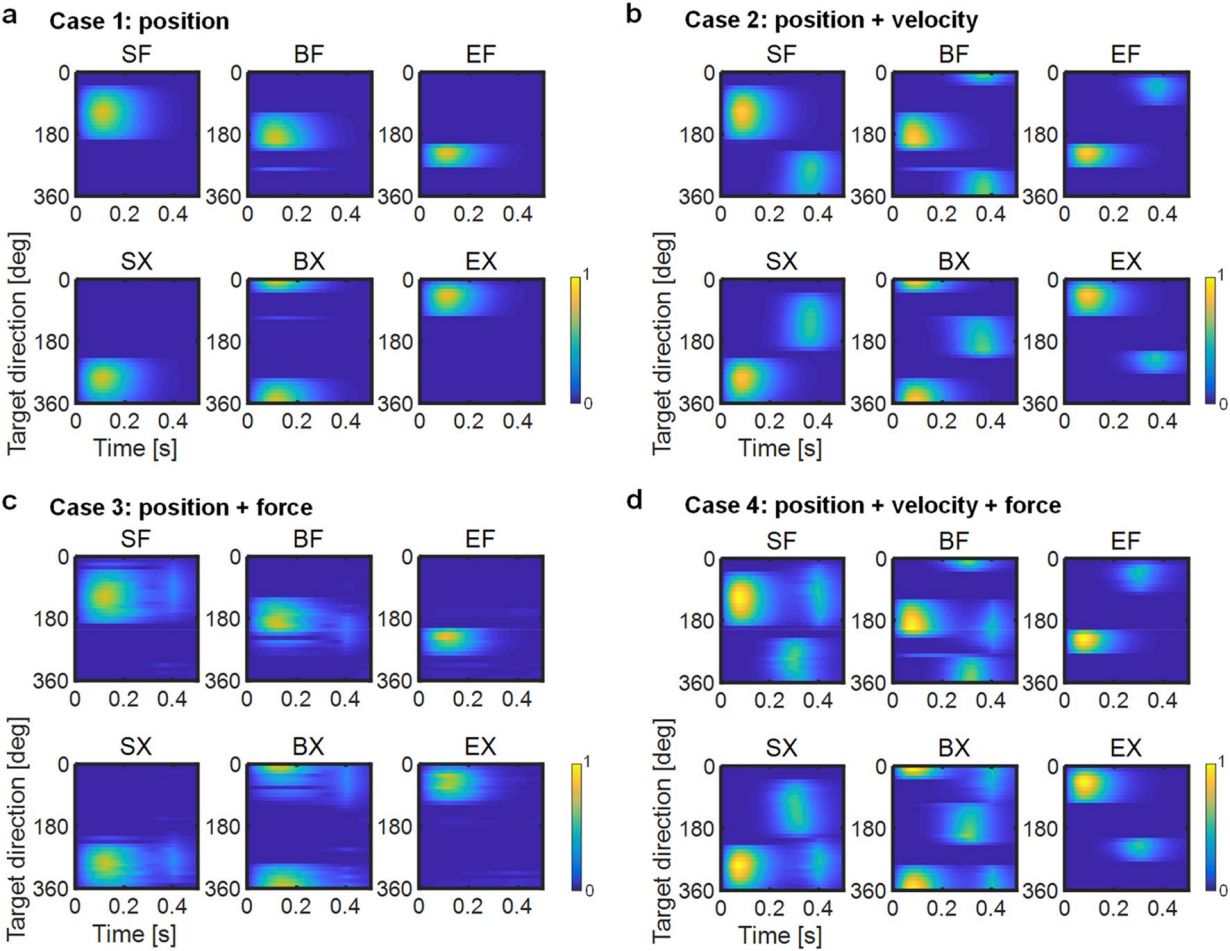
Muscle activation patterns plotted as a function of time and target direction. The target direction is counter-clockwise from the right. Cases 1–4 are shown in (**a**)–(**d**), respectively. Values are normalised using the highest activation level in each muscle across all cases. Muscle activation levels were normalised by the highest activation level in each muscle across all cases: SF, 0.32; SX, 0.35; BF, 0.24; BX, 0.23; EF, 0.28; EX, 0.29.

Our model showed that the hand pathways varied slightly, forming curves or almost-straight lines in all cases, according to direction (Fig. 2, left column). However, the movement parameters and muscle activation patterns (Fig. 3) showed distinct specifications according to the cost functions. Although the muscles were selectively activated according to the target direction, which was defined as a counter-clockwise direction from the right (i.e. the *x*-direction), regardless of the case following the first muscle activation (AG1), the second (ANT) and third (AG2) bursts of the muscles varied temporally among cases.

### Movement parameters

**Figure 4:**
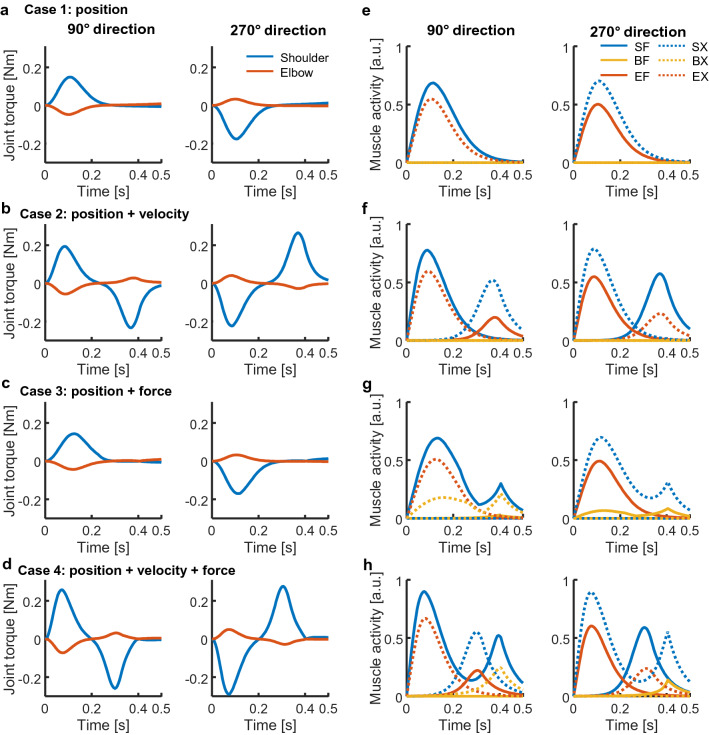
Example of joint torques and muscle activities for forward (90°) and backward directions (270°). Cases 1–4 are in rows from top to bottom, respectively. (**a**–**d**) Joint torques profiles. (**e**–**h**) Muscle activities.

In Case 1, the hand passed through on the targets and the speed did not converge on zero at the end of movement (i.e. 400 ms); the joint torques peaked at the onset of movement to generate the hand force triggering the movement (Figs. 2a, 4a). Then, the hand force is shown as the root mean square (RMS) computed as $\sqrt {f_{x}^{2} + f_{y}^{2} }$, where *f*_*x*_ and *f*_*y*_ are the lateral and longitudinal forces generated at the hand in the horizontal plane, respectively (Fig. 2). The joint torques and hand force then diverged slightly at the movement end because a passive tensile force was generated by stretching the muscles. In Case 2, the hand stopped on the targets and the speed was almost set to zero at the movement end, although slight motions were observed after the movement end (Figs. 2b, 4b). The hand force and joint torques showed biphasic peaks. The second peak of the joint torques was generated in the direction opposite to the movement, thus decelerating the movement and setting the hand speed to zero at the movement end. However, the hand force and joint torques did not converge at zero at the movement end; instead, they gradually decreased. In Case 3, the hand passed through the targets and the speed was not zero at the movement end time, similar to the findings in Case 1; furthermore, the hand force and joint torques converged on zero at this time in contrast to Case 1 (Figs. 2c, 4c). In Case 4, the hand stopped on the targets and the speed was close to zero with gradual curves, forming clear bell-shaped profiles (Figs. 2d, 4d). Although the hand force and joint torques showed biphasic peaks similar to the findings in Case 2, they converged on zero at the movement end.

### Muscle activation patterns

In Case 1, agonist muscles were activated once to initiate movement (Figs. 3a, 4e). Then, the monoarticular elbow muscles EF and EX showed no activation. In Case 2, activation of the agonist and antagonist muscles alternated similarly to AG1 and ANT burst, occurring immediately after the beginning and at the end of movement, respectively (Figs. 3b, 4f). The antagonist muscles might contribute torque in the direction opposite to the movement to decelerate the movement. However, EF and EX were activated only as antagonist muscles synchronising with the biarticular muscles BF or BX. In Case 3, the agonist muscles were activated twice, similar to bursts of AG1 and AG2 (Figs. 3c, 4g). The second activations might suppress the development of hand force and joint torques at the end of movement. Muscles EF and EX were weakly activated to supplement BF or BX. In Case 4, muscle activations showed a triphasic pattern resembling the superposition of activations in Cases 2 and 3, such that the agonist and antagonist muscles were activated in an alternating manner; however, the agonist muscles were occasionally activated twice (Fig. 3d). Notably, when the SF muscle acted as an agonist (e.g. 90° movement direction; Fig. 4h: left), it was activated twice, at the start and end of movement; it was activated only once in the middle of movement when it was an antagonist muscle (e.g. 270° movement direction; Fig. 4h: right). The later activation of agonist muscles may contribute to movement stabilisation because the later torque observed in Case 2, which the antagonist muscles generated, may induce unnecessary movement after reaching the target. Thus, the additional torque generated by the agonist muscles was required to counteract opposite torques and stabilise movement after completing the task.

The costs of the position, velocity, and force corresponded to AG1, ANT, and AG2, respectively. Thus, Case 4 could only reproduce the triphasic muscle activation pattern.

### Stabilisation control

Our results suggest that the force cost reactivates the agonist muscle to counteract the breaking torques for stabilisation. An alternate hypothesis is that to achieve a reaching movement within a certain timeframe, the stability of the terminal kinematic state (i.e. position, or position and velocity) must be optimised after the movement phase, but not to minimise the terminal cost solely at the movement end. To investigate this hypothesis, we defined two extra cost functions for stabilisation control as shown in Eq. (11).

**Figure 5:**
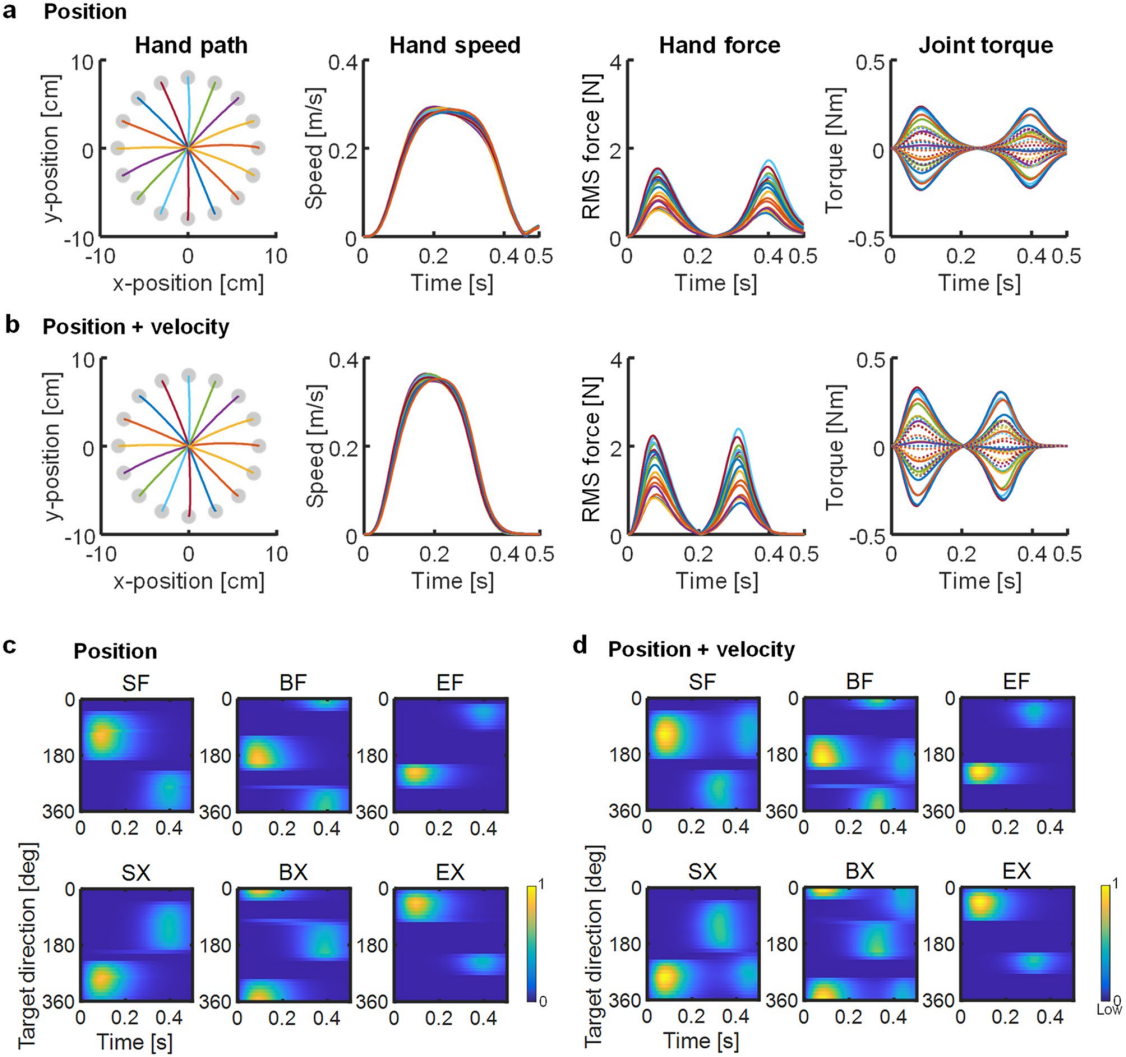
Stabilisation control. Controls of position cost and two position and velocity costs are shown in (**a**, **c**) and (**b**, **d**), respectively. (**a**, **b**) Centre-out movement for 16 targets. The format is identical to Fig. 2. (**c**, **d**) Muscle activation pattern. The format is identical to Fig. 3. Muscle activation levels were normalised by the highest activation level in each muscle across both cases: SF, 0.31; SX, 0.33; BF, 0.23; BX, 0.23; EF, 0.27; EX, 0.28.

Although the cost function in Eq. (11) showed the biphasic muscle pattern present in Case 2 (Fig. 5c), the cost function in Eq. (12) reproduced the triphasic muscle pattern present in Case 4 (Fig. 5d). Accordingly, we could not reject the hypothesis of stabilisation control represented by Eq. (12). However, stabilisation control may regulate a cost term differential because the stabilisation of Eq. (11), which adopts only the position cost, decreased the hand speed in a manner similar to Case 2 (Fig. 5a). Therefore, we presume that the stabilisation control of Eq. (12) suppressed hand acceleration during the stabilisation period (Fig. 5b), and it played a role similar to the force cost in Case 4.

### Effects of movement duration on muscle activation patterns

**Figure 6:**
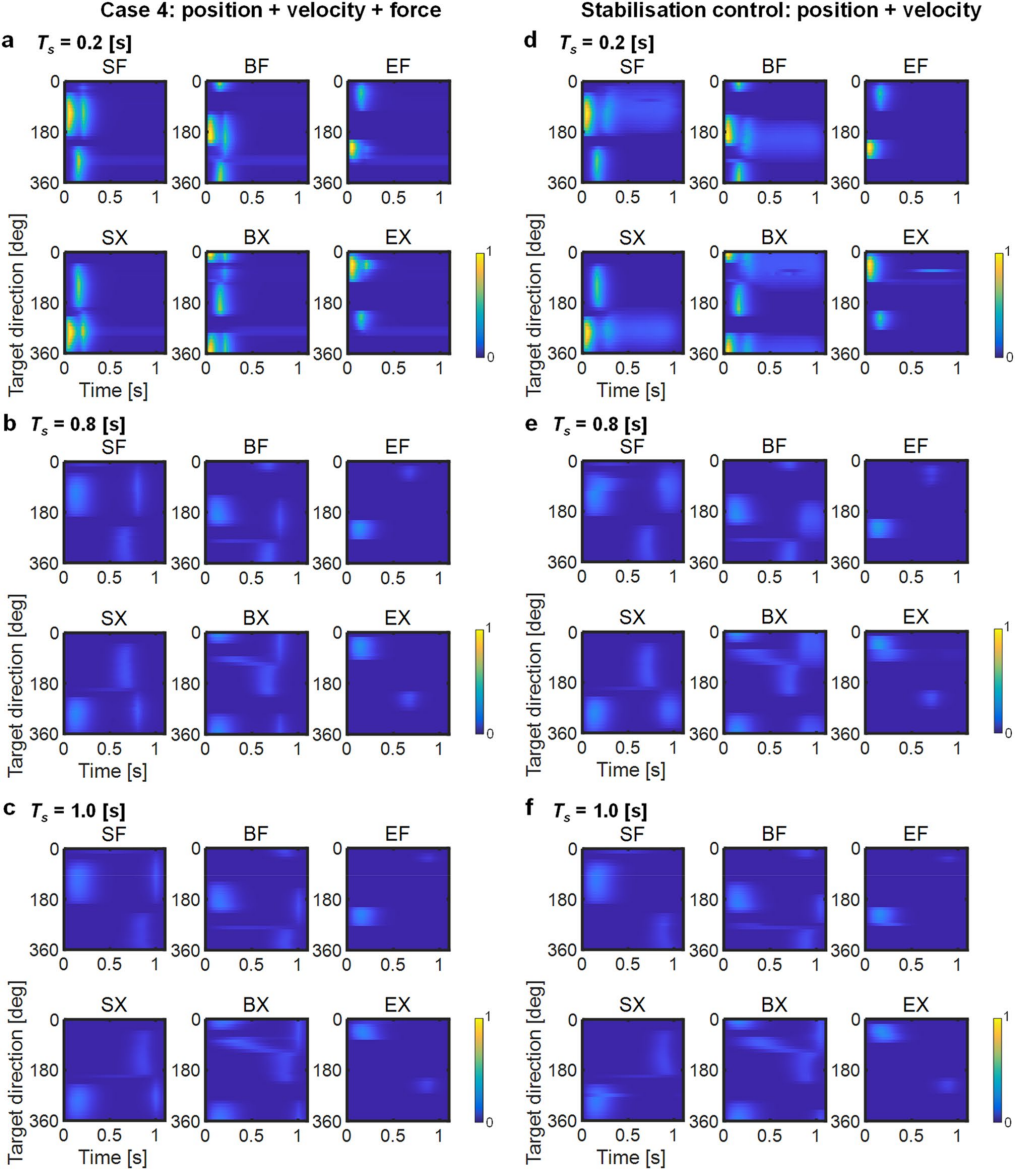
Changes of muscle activation patterns in relation to movement duration. Muscle activation patterns of movement times at 0.2, 0.8, and 1.0 s are shown from top to bottom, respectively. Case 4 and the stabilisation control are shown in (**a**–**c**) and (**d**–**f**), respectively. Values were normalised using the highest activation level in each muscle across all movement durations, (**a**–**c**) SF, 0.64; SX, 0.68; BF, 0.45; BX, 0.45; EF, 0.51; EX, 0.54, (d–f) SF, 0.57; SX, 0.61; BF, 0.42; BX, 0.42; EF, 0.47; EX, 0.48.

As the movement duration was shorter and the velocity of movement increased, the muscle activities were strengthened while the activation duration decreased. Slower movements were associated with prolonged activation from the agonist muscle with little or no antagonist activity, although the agonist bursts twice^38^. We evaluated the effects in Case 4 and the stabilisation control adopting the position and velocity costs to change their movement durations to 0.2 s, 0.8 s, and 1.0 s under the simulation time at 1.1 s (Fig. 6).

The triphasic pattern in Case 4 was consistent, regardless of movement duration, although the bursts were weakened under prolonged duration when the movement duration was long (Fig. 6a–c). In the stabilisation control, the muscle activation pattern changed from triphasic to biphasic for the shoulder muscles (SF and SX) at the movement end 1.0 s (Fig. 6f), although it was a similar effect to Case 4 (Fig. 6d–f). Deformation of the muscle activation pattern occurred because of a decrease in the antagonist muscle burst at long movement durations. The agonist muscle SF and SX (i.e. AG2) were not required to negate the breaking torques induced by the antagonist muscle ANT. However, in Case 4, the agonist muscles were required to compensate the passive tensile force of the stretched muscles according to force cost, regardless of movement duration.

## Discussion

This study investigated the requirements for deriving muscle activation patterns, in which antagonistic muscle pairs show alternating bursts with triphasic shapes. To achieve this aim, we carried out simulations of arm movements and applied four types of cost function, considering the end-point position, velocity, and force requirements under minimisation of the neural input. To summarise the results, the position, velocity, and force costs were found to play following roles: (1) the position cost led to the activation of the first agonist muscle, triggering movement; (2) the velocity cost activated the antagonist muscles and generated braking torques to decelerate the movement; and (3) the force cost reactivated the agonist muscle to negate the breaking torques and the passive tensile force of the stretched muscles, providing stabilisation. Other stabilising cost functions of the terminal position and velocity after the movement phase also produced the triphasic muscle pattern. However, the pattern was deformed at long movement duration, while the cost function adopting the position, velocity, and force exhibited a consistent pattern, regardless of movement duration. Taken together, our results suggest that triphasic muscle activation is induced by the costs of the position, velocity, and force at the movement end.

**Figure 7:**
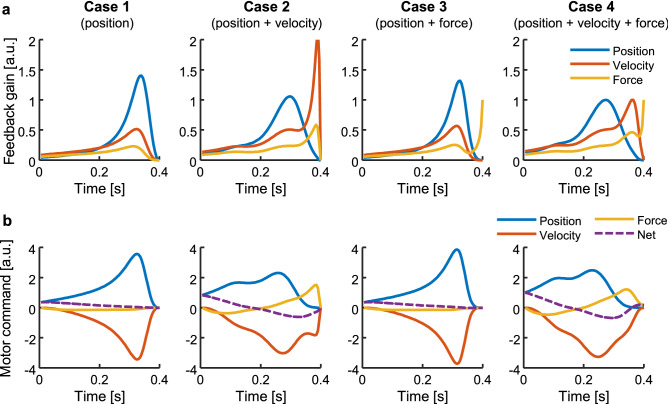
Typical feedback gains of the optimal feedback control (OFC) model and the contributions to motor command. The optimal feedback gains were analytically computed for a linear system to model hand movements as mass-point dynamics. Each case (Cases 1–4) corresponds to the same formation of the cost function in the above results. (**a**) Feedback gains. Each value of the position, velocity, and force gains was normalised to the values of Case 4. (**b**) Contributions of the feedback terms to the net motor command. Each value was normalised to the net motor command of Case 4.

Our control methodology was only approximately optimal, given the challenge of solving the optimal control problem analytically in nonlinear systems such as the musculoskeletal system. According to the original OFC model for a linear dynamics plant whose state variable **x**(*t*) comprises the position, velocity, and force^16^, the motor command is represented as·${\mathbf{u}}\left( t \right) = {\mathbf{K}}\left( t \right) \cdot {\hat{\mathbf{x}}}\left( t \right)$, where **K**(*t*) is the feedback gain and ${\hat{\mathbf{x}}}\left( t \right)$ is the estimated state integrating the prediction of the internal model (i.e. feedforward model) with the sensory feedback similar to a Kalman filter. Because the Kalman filter allowed a delay in the feedback depending on the physiological systems, the feedback delays did not directly affect feedback gains. The components of the feedback gain for the position, velocity, and force peaked at various times, corresponding to the cost functions of Cases 1–4 (Fig. 7a). Simultaneous peaks were observed in Case 1; two peaks were observed in Cases 2 and 3; and the position, velocity and force peaked in turn in the early, mid, and late phases of movement in Case 4. Then, the position gain served to initiate movements that generated positive motor command, and the velocity and force gains contributed to decelerate and stabilise the movement (Fig. 7b), thus supporting our hypothesis regarding the roles of the cost terms.

This approach may close the gap between ideal arm movements in the horizontal plane and actual nonlinear dynamics in neurophysiological systems. Indeed, it has been difficult to acquire optimal solutions for more complex and realistic motor control, such as multi-dimensional motions that recruit numerous muscles; however, OFC may be provided by a neural substrate in M1, integrating joint motion information into joint torque for fast feedback control^39,40^. Thus, trajectory planning in kinematics and the muscle activation pattern may be generated in separate steps^41,42^. Based on this idea, a hierarchical control framework has been suggested, which divides the control problem into high-level and low-level controllers^42,43^. The high-level controller is designed to capture the main features of the complex high-dimensional plant dynamics, although it exhibits reduced dimensionality, similar to a mass-point dynamics system. The low-level controller generates arm configurations and muscle activations to exactly match the high-level controls, while concurrently satisfying biological constraints. Although the solutions of this framework are essentially identical to the solutions acquired by the approach used in this work, the optimal solution for this system was acquired and we did not apply any external load; therefore, it could potentially be used to understand how neural systems handle natural biological motions.

Several studies in patients with motor disorders have demonstrated that the basal ganglia may have a role in scaling the size of AG1, reinforcing voluntary command and inhibiting inappropriate EMG activity^6^. Therefore, we assumed that in the basal ganglia module, the positional cost weight *w*_*p*_ is related to AG1. The cerebrum may regulate the other cost weights *w*_*v*_ and *w*_*f*_ to balance *w*_*p*_, because the cerebellum plays a role in timing the voluntary bursts of ANT and AG2. Indeed, cerebellar patients are unable to perform accurate movements^44^. This deficit is known as dysmetria, which is a lack of coordination of movement that results in overshooting or undershooting a target during reaching tasks.

Dehghani and Bahrami suggested that arm movements are planned with some principal patterns of muscle synergies; the plans can be divided into relatively few phases to reduce the dimension of the control space^45,46^. Furthermore, Sakaguchi et al. proposed a computational model in which the brain adaptively divides the continuous-time axis into discrete segments and executes feedforward control in each segment to allow sensorimotor delays^47^. However, the OFC realised control of the muscle synergies through feedback gains, and the segmentation of motor execution may have been identified as steady-state feedback gains computed by a model predictive control under the framework of the OFC^23^.

In previous studies that used similar muscle models^25,35^, the tensile force of the passive elastic component was excluded from the muscle model (i.e. *F*_*PE*2_ in Eq. (8)), despite the size of the force. We assume that this was excluded because of parameter selection sensitivity for the muscle model, such as the moment arms, optimal muscle lengths, and optimal joint angles, which affect the muscle length and generate a tensile force without muscle activation. In our model, the tensile force requires initial muscle activation to maintain the initial position before movement onset; this affects the hand trajectories, causing marked distortion. Thus, we set the initial hand position to an equilibrium point balancing the forces, ensuring that the initial muscle activations remain at zero. With such sensitive effects, it is important to investigate how movement can be generated and controlled. Previous studies suggested that the neurons are optimised for physical mechanics^23–25^. However, if individuals have learned fine motor tasks once, such learnings tend to persist, regardless of whether they are clearly suboptimal^48^. Subsequently, individuals learn to overcome real and virtual changes in their biomechanics, but prefer to rescale their prior motor habits, rather than recomputing to optimise the control policy^49^. The prior motor habits may be learned from muscle activation patterns generated by lower sensorimotor circuitry that is functionally suboptimal. However, we ignored the lower sensorimotor circuitry, which is a limitation of our model. Recently, a model of the spinal circuitry and musculoskeletal system was developed^50^, which may be useful for studying habitual motor control systems.

The OFC framework has another potential limitation. The model cannot predict movement duration, but requires this duration as an input. Indeed, the amplitudes and timings of the muscle activities and feedback gains are directly related to movement time and velocity. According to the findings in a previous study^51^, the movement duration affects the cost function in optimal control, through a mechanism equivalent to reward delay; this is because the passage of time reduces the value of a reward in the human brain^52^. Shadmehr et al. suggested a cost function introducing the cost of time as a hyperbolic function to the OFC-like cost function^51^. However, the cost of time is dependent on parameters in the hyperbolic function of the model, and the cost appears to be influenced by several factors (e.g. life span^53^).

In summary, the results of this study imply that a triphasic muscle activation pattern can be predicted by an optimal control mechanism (e.g. by adopting an OFC-like cost function). Furthermore, the costs of position, velocity, and force requirements may be critical parameters for the physiological control of movements; they correspond to the triggering, braking, and stabilising of movement, respectively.

## Methods

We considered the monkey’s arm to be a two-joint arm composed of the shoulder and elbow joints. The joint angles were defined as vector **θ = **[*θ*_1_, *θ*_2_]^*T*^, where *θ*_1_ and *θ*_2_ indicate the shoulder and elbow variables, respectively. Suppose joint torque **τ** ∈ *R*^2^, the dynamics of the monkey’s arm in horizontal plane are denoted by

1 $$ {{\varvec{\uptau}}} = {\mathbf{M}}({{\varvec{\uptheta}}}) \cdot {\ddot{\mathbf{\varvec{\uptheta}}}} + {\mathbf{C}}({{\varvec{\uptheta}}},\;{\dot{\mathbf{\varvec{\uptheta}}}}) + {\mathbf{D}} \cdot {\dot{\mathbf{\varvec{\uptheta}}}},$$

where **M**(·) ∊ *R*^2×2^, **C**(·) ∊ *R*^2^, and **D** ∊ *R*^2×2^ are the inertia matrix, Coriolis force vector, and viscosity matrix, respectively, and are given by

$$ {\mathbf{M}}({{\varvec{\uptheta}}}) = \left[ {\begin{array}{*{20}l} {s_{1} + 2s_{2} \cos \theta_{2} } \hfill & {s_{3} + s_{2} \cos \theta_{2} } \hfill \\ {s_{3} + s_{2} \cos \theta_{2} } \hfill & {s_{3} } \hfill \\ \end{array} } \right], $$

$${\mathbf{C}}({\mathbf{\varvec{\uptheta}}},{\dot{\mathbf{\varvec{\uptheta}}}}) = \left[ {\begin{array}{*{20}l} {\begin{array}{*{20}l} { - \dot{\theta }_{2} (2\dot{\theta }_{1} + \dot{\theta }_{2} )} \hfill \\ {\dot{\theta }_{1}^{2} } \hfill \\ \end{array} } \hfill \\ \end{array} } \right] \cdot s_{2} \sin \theta _{2} ,{\mkern 1mu} {\mkern 1mu} {\mkern 1mu} {\mathbf{D}} = \left[ {\begin{array}{*{20}l} {d_{{11}} } \hfill & {d_{{12}} } \hfill \\ {d_{{21}} } \hfill & {d_{{22}} } \hfill \\ \end{array} } \right],$$

$$ s_{1} = I_{1} + I_{2} + m_{2} l_{1}^{2} ,\,\,\,s_{2} = m_{2} l_{1} l_{g2} ,\,\,\,s_{3} = I_{2} . $$

**Figure 8:**
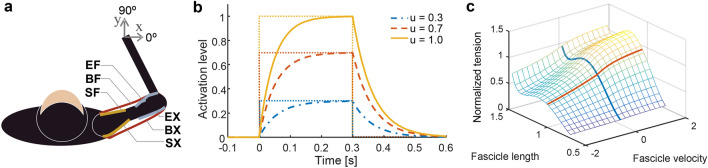
Simulation model. (**a**) Allocation of muscles in the two-link, six-muscle arm model. The SF models the pectoralis major, coracobrachialis, and deltoid anterior muscles. The SX models the posterior and middle deltoid muscles. The BF models the long and short biceps muscles. The BX models the long triceps muscle. The elbow flexor (EF) models the brachialis, brachioradialis, and extensor carpi radialis longus muscles. The elbow extensor (EX) models the lateral and medial triceps muscles. (**b**) Muscle activation dynamics. (**c**) Length–velocity–tension curve of the muscle model.

They are represented by the link parameters: mass *m*_*i*_, length *l*_*i*_, distance from the joint centre of mass *l*_*gi*_, moment of inertia *I*_*i*_, joint friction *d*_*i1*_, *d*_*i2*_ (*i* = 1, upper arm; *i* = 2, forearm). The parameters are shown in Table 1, and were estimated from our measurements of a Japanese macaque. Because many muscles act on the arm in the horizontal plane, we modelled only two degrees of freedom actuated by six muscle groups: SF, shoulder flexor; SX, shoulder extensor; BF, biarticulate flexor; BX, biarticulate extensor; EF, elbow flexor; and EX, elbow extensor (Fig. 8a). The joint torque is a function of its moment arms **A** ∈ *R*^2×6^ and the muscle tension vector **T** = [*T*_1_, *T*_2_, *ˑˑˑ*, *T*_6_]^*T*^, and it is given by **τ = A**·**T**. The moment arm is defined as the perpendicular distance from the muscle line of action to the joint centre of rotation, given by

2 $$ {\mathbf{A}} = \left[ {\begin{array}{*{20}c} {1.5} & { - 1.5} & 0 & 0 & {1.5} & { - 1.5} \\ 0 & 0 & {1.5} & { - 1.5} & {1.5} & { - 1.5} \\ \end{array} } \right]/100\,[{\text{m}}], $$

**Table 1:** Link parameters.

Link	*m*_*i*_ (kg)	*l*_*i*_ (m)	*l*_*gi*_ (m)	*I*_*i*_ (kg m^2^)	*d*_*i*1_ (N m s/rad)	*d*_*i*2_
*i* = 1	0.3	0.15	0.07	5.0 × 10^–3^	5.0 × 10^–3^	2.5 × 10^–3^
*i* = 2	0.3	0.21	0.12	9.0 × 10^–3^	2.5 × 10^–3^	5.0 × 10^–3^

The *j*th muscle activation *a*_*j*_ (*j* = 1, 2, …, 6) is not equal to the instantaneous neural input *u*_*j*_; instead, it is generated by passing *u*_*j*_ through a filter that describes the calcium dynamics modelled with a first-order nonlinear filter^54^:

3 $$ \dot{a}_{j} = \frac{{u_{j} - a_{j} }}{{f(u_{j} ,\;a_{j} )}}, $$

where

$$ f(u_{j} ,\;a_{j} ) = \left\{ {\begin{array}{*{20}l} {t_{deact} + u_{j} (t_{act} - t_{deact} )} \hfill & {u_{j} > a_{j} } \hfill \\ {t_{deact} } \hfill & {u_{j} \le a_{j} } \hfill \\ \end{array} } \right.. $$

The time constant parameters were set as *t*_*act*_ = 0.05 [s] and *t*_*deacct*_ = 0.066 [s] because the input-dependent activation dynamics are faster than the constant deactivation dynamics (Fig. 8b).

Mammalian muscles have remarkable scaling properties, meaning that all are similar after proper normalisation: length is expressed in units of ${\text{L}}_{{\text{j}}}^{0}$ the length at which the maximum isometric force is generated^55^, and velocity is expressed in units ${\text{L}}_{{\text{j}}}^{0}$ per second^56^. Thus we assume that the units are unified across all muscles as *L*^0^ = 0.08 [m], and we denote a normalised muscle length *L*_*j*_ as follows:

4 $$ L_{j} = 1 + {{A_{j}^{T} ({{\varvec{\uptheta}}}_{j}^{0} - {{\varvec{\uptheta}}})} \mathord{\left/ {\vphantom {{A_{j}^{T} ({{\varvec{\uptheta}}}_{j}^{0} - {{\varvec{\uptheta}}})} {L^{0} }}} \right. \kern-\nulldelimiterspace} {L^{0} }}, $$

where *A*_*j*_ is the *j*th row vector of **A**, ${{\varvec{\uptheta}}}_{j}^{0} \in R^{2}$ is the optimal joint angle vector of the *j*th muscle for generating the maximal torque in the shoulder and elbow, respectively. Then the muscle tension *T*_*j*_ is given to scale the unit-less tension by the absolute muscle force of the physiological cross-sectional area (PCSA) to yield the physical tension^57^:

5 $$ T_{j} = F_{a} \cdot P \cdot \overline{T}_{j} , $$

where *F*_*a*_ is the absolute muscle force and is set to *F*_*a*_ = 32 [N/cm^2^] based on measurements in monkeys^58^, and *P* is the PCSA that is assumed to be uniform across muscles as *P* = 10 [cm^2^]. According to a model of mammalian skeletal muscle^56,59^, the unit-less muscle tension is produced by a nonlinear muscle model composed of the functions of contractile element *F*_*CE*_(·) and passive elastic element *F*_*PE*_(·):

6 $$ \overline{T}_{j} = F_{CE} (a_{j} ,\;L_{j} ,\;\dot{L}_{j} ) + F_{PE} (L_{j} ), $$

where

7 $$ F_{CE} (a_{j} ,\;L_{j} ,\;\dot{L}_{j} ) = A_{f} (a_{j} ,\;L_{j} ) \cdot F_{L} (L_{j} ) \cdot F_{V} (L_{j} ,\;\dot{L}_{j} ), $$

8 $$ F_{PE} (a_{j} ,\;L_{j} ) = F_{PE1} (L_{j} ) + A_{f} (a_{j} ,\;L_{j} ) \cdot F_{PE2} (L_{j} ). $$

Here, *A*_*f*_(·), *F*_*L*_(·), *F*_*V*_(·) are the functions of the activation-frequency relationship, the tetanic force–length relationship, and the tetanic force–velocity relationship, respectively. The passive elastic force is represented by two separate functions, *F*_*PE*1_(·) and *F*_*PE*2_(·), which exert a tensile force and resist compression force, respectively. They are defined as follows:

$$ A_{f} (a_{j} ,\;L_{j} ) = 1 - \exp \left[ { - \left\{ {\frac{{a_{j} }}{{0.56\left( {2.12 + 3.31({1 \mathord{\left/ {\vphantom {1 {L_{j} }}} \right. \kern-\nulldelimiterspace} {L_{j} }} - 1)} \right)}}} \right\}^{{2.12 + 3.31({1 \mathord{\left/ {\vphantom {1 {L_{j} }}} \right. \kern-\nulldelimiterspace} {L_{j} }} - 1)}} } \right], $$

$$ F_{L} (L_{j} ) = \exp \left( { - \left| {\frac{{L_{j}^{1.55} - 1}}{0.81}} \right|^{2.12} } \right), $$

$$ F_{V} (L_{j} ,\;\dot{L}_{j} ) = \left\{ {\begin{array}{*{20}l} {\frac{{ - 7.39 - \dot{L}_{j} }}{{ - 7.39 - (3.21 - 4.17L_{j} )\dot{L}_{j} }}} \hfill & {\dot{L}_{j} \le 0} \hfill \\ {\frac{{1.05 + 1.53\dot{L}_{j} }}{{1.05 + \dot{L}_{j} }}} \hfill & {\dot{L}_{j} > 0} \hfill \\ \end{array} } \right., $$

$$ F_{PE1} (L_{j} ) = 0.15\log \left\{ {\exp \left( {\frac{{L_{j} - 1.54}}{0.059}} \right) + 1} \right\}, $$

$$ F_{PE2} (L_{j} ) = \min \left[ { - 0.02\left\{ {\exp \left( {18.7(L_{j} - 0.79)} \right) - 1} \right\},\;\;0} \right]. $$

The dependence of force on the length and velocity of a muscle are often referred to as the force–length and force–velocity curves, respectively (Fig. 8c). The muscle model parameters are shown in Table 2; these were assigned based on the results of anatomical measurements of macaque monkeys^58,60^.

**Table 2:** Optimal joint angles of each muscle.

		*j* = 1	*j* = 2	*j* = 3	*j* = 4	*j* = 5	*j* = 6
$\frac{180}{\pi }{{\varvec{\uptheta}}}_{j}^{0}$	(deg)	15	5	–^a^	–^a^	15	5
		–^a^	–^a^	90	110	100	100

^a^Does not affect the muscle length regardless of the value, because the moment arms are set to zero to avoid transforming the effects.

### Approximately optimal feedback control

We transformed the two-joint six-muscle model into a state–space model. The control object was denoted by the state vector **x** ∊ *R*^10^ as

9 $${\mathbf{x}} = \left[ {\begin{array}{*{20}c} {{\varvec{\uptheta}}} \\ {{\dot{\mathbf{\varvec{\uptheta}}}}} \\ {\mathbf{a}} \\ \end{array} } \right],$$

where **a** = [*a*_1_, *a*_2_, …, *a*_6_]^*T*^ is the muscle activation vector. Here, we define the state vector at time *t* as **x**(*t*). Then, the dynamics of the musculoskeletal arm model can be written as a state–space equation described as.

$${\dot{\mathbf{x}}}(t) = F({\mathbf{x}}(t)) + G({\mathbf{x}}(t)) \cdot {\mathbf{u}}(t).$$

The nonlinear functions *F*(·) and *G*(·) are defined for descriptive purposes to represent the dynamics in an affine form. In practice, they are given as locally linearised forms around each state at time *t* to obtain an approximately OFC law. The motor command **u**(*t*) is given by **u**(*t*) = $\overline{\mathbf{u} }$(*t*) + **K**(*t*)∙($\overline{\mathbf{x} }$(*t*) − **x**(*t*)), where $\overline{\mathbf{u} }$(*t*) is an open-loop control component, **K**(*t*) is a feedback gain, and $\overline{\mathbf{x} }$(*t*) is a nominal trajectory. The parameters are computed iteratively to optimise $\overline{\mathbf{u} }$(*t*) using the Levenberg–Marquardt algorithm to minimise the following cost function:

10 $$ q_{1} = w_{p} \left\| {{\mathbf{p}}(T_{s} ) - {\mathbf{p}}^{*} } \right\|^{2} + w_{v} \left\| {{\mathbf{v}}(T_{s} )} \right\|^{2} + w_{f} \left\| {{\mathbf{f}}(T_{s} )} \right\|^{2} + \int_{0}^{T} {\left\| {{\mathbf{u}}(t)} \right\|^{2} dt} , $$

where *T*_*s*_ and *T* are the terminal time of movement and simulation duration, respectively. The iterative optimisation process finally brings an outcome converging **K**(*t*)* ≈* **0** and $\overline{\mathbf{x} }$(*t*) *≈ ***x**(*t*). Note that we set the times *T*_*s*_ = 0.4 [s] and *T* = 0.5 [s] as default, and *T*_*s*_ = 0.2, 0.8, or 1.0 [s] and *T* = 1.1 [s] for evaluation of movement time effect. The vectors **p**(*T*_*s*_) ∊ *R*^2^, **v**(*T*_*s*_) ∊ *R*^2^, and **f**(*T*_*s*_) ∊ *R*^2^ are the end-point position, velocity, and force in Cartesian space at the movement end *T*_*s*_, respectively. They are calculated from the joint angles, angular velocities, and torques as follows:

$$\begin{aligned} {\mathbf{p}}(t) & = \left[ {\begin{array}{*{20}c} {l_{1} \cos \theta_{1} (t) + l_{2} \cos (\theta_{1} (t) + \theta_{2} (t))} \\ {l_{1} \sin \theta_{1} (t) + l_{2} \sin (\theta_{1} (t) + \theta_{2} (t))} \\ \end{array} } \right], \\ {\mathbf{v}}(t) & = {\mathbf{J}}(t) \cdot {\dot{\mathbf{\varvec{\uptheta}}}}(t), \\ {\mathbf{f}}(t) & = ({\mathbf{J}}(t)^{T} )^{ - 1} \cdot {{\varvec{\uptau}}}(t), \\ \end{aligned}$$

where **J**(*t*) ∈ *R*^2×2^ is the Jacobian matrix

$$ {\mathbf{J}}(t) = \left[ {\begin{array}{*{20}c} { - l_{1} \sin \theta_{1} (t) - l_{2} \sin (\theta_{1} (t) + \theta_{2} (t))} & { - l_{2} \sin (\theta_{1} (t) + \theta_{2} (t))} \\ {l_{1} \cos \theta_{1} (t) + l_{2} \cos (\theta_{1} (t) + \theta_{2} (t))} & {l_{2} \cos (\theta_{1} (t) + \theta_{2} (t))} \\ \end{array} } \right]. $$

In addition, p* is a target position in Cartesian space, and *w*_*p*_, *w*_*v*_, and *w*_*f*_ are cost weights of the position, velocity, and force requirements at the movement end, respectively. On the right-hand side of Eq. (10), the first, second, and third terms evaluate the requirements of the end-point position, velocity, and force, respectively, at the end of movement in achieving the desired state, whereby the end-point is close to the target position with zero values for the end-point velocity and force. The fourth term, which is the sum of the squares of the neural inputs during the movement, evaluates the metabolic cost of the neural input. This plays a role in minimising the end-point variance at the movement end under the condition of motor noise, known as signal-dependent noise^61^. However, the noise was not incorporated into this model for simplification, enabling us to focus on the muscle activities excluding external effects.

### Cost weight parameters

We simulated four situations requiring movement under minimised neural input by balancing the cost weights in Eq. (10) (Table 3). These were determined heuristically to achieve the task. We confirmed that the results of this model were robust under parameter changes by performing a sensitivity analysis (see Supplementary Note 1).

**Table 3:** Cost weight parameters.

	*w*_*p*_ (position)	*w*_*v*_ (velocity)	*w*_*f*_ (force)
Case 1	1000	0	0
Case 2	1000	100	0
Case 3	1000	0	10
Case 4	1000	100	10

### Stabilisation control

We examined an alternate hypothesis that stabilises the terminal position, or position and velocity, after the movement phase; however, it does not minimise only the terminal cost at the movement end. Thus, we additionally defined following two cost functions for stabilisation control:

11 $$ q_{2} = \frac{1}{{T - T_{s} }}\int_{{T_{s} }}^{T} {w_{p} \left\| {{\mathbf{p}}(t) - {\mathbf{p}}^{*} } \right\|^{2} dt + \int_{0}^{T} {\left\| {{\mathbf{u}}(t)} \right\|^{2} dt} } , $$

and

12 $$ q_{3} = \frac{1}{{T - T_{s} }}\int_{{T_{s} }}^{T} {\left( {w_{p} \left\| {{\mathbf{p}}(t) - {\mathbf{p}}^{*} } \right\|^{2} + w_{v} \left\| {{\mathbf{v}}(t)} \right\|^{2} } \right)dt + \int_{0}^{T} {\left\| {{\mathbf{u}}(t)} \right\|^{2} dt} } . $$

Then, we set the default time at the movement end and stabilisation period as 0.5 s and 0.1 s, respectively (i.e. *T*_*s*_ = 0.4 [s], *T* = 0.5 [s]). To evaluate effects of changes in the movement duration, the movement duration was set to 0.2 s, 0.8 s, or 1.0 s under the simulation time equal to 1.1 s (i.e. *T*_*s*_ = 0.2, 0.8, 1.0 [s]; *T* = 1.1 [s]).

## Supplementary Information

Supplementary Information 1.

Supplementary Video 1.

## Data Availability

The MATLAB (MathWorks, Natick, MA, USA) codes and data sets that support the findings of this study are available at GitHub (https://github.com/yuki-ueyama/Muscle-Activation-Pattern).
