# Author Correction: Costs of position, velocity, and force requirements in optimal control induce triphasic muscle activation during reaching movement

**DOI:** 10.1038/s41598-021-98291-3

**Published:** 2021-09-13

**Authors:** Yuki Ueyama

**Affiliations:** Department of Mechanical Engineering, National Defense Academy of Japan, Yokosuka, Kanagawa Japan

Correction to: *Scientific Reports* 10.1038/s41598-021-96084-2, published online 19 August 2021

The original version of this Article contained an error in Figure 4 where the line graphs were incorrect in panels e-h. The original Figure 4 and accompanying legend appear below.

**Figure 4:**
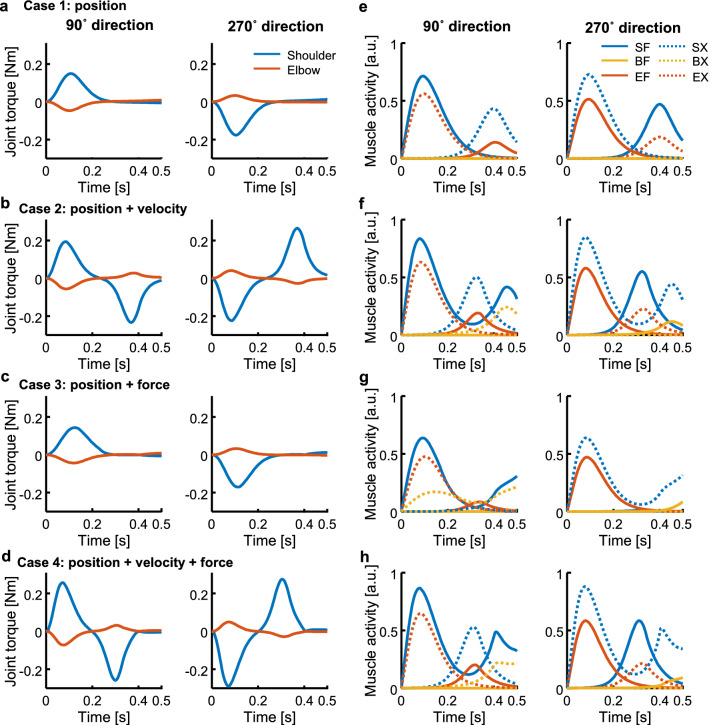
Example of joint torques and muscle activities for forward (90°) and backward directions (270°). Cases 1–4 are in rows from top to bottom, respectively. (**a**–**d**) Joint torques profiles. (**e**–**h**) Muscle activities.

The original Article has been corrected.

