# Supplementary material for: Costs of position, velocity, and force requirements in optimal control induce triphasic muscle activation during reaching movement
Source: Sci Rep. 2021 Aug 19;11:16815. doi: 10.1038/s41598-021-96084-2 (PMC8376873; doi:10.1038/s41598-021-96084-2)
Supplement: Supplementary file 1 — Supplementary Information 1. [file 41598_2021_96084_MOESM1_ESM.pdf]

# Supplementary Information

## Supplementary Note

The Supplementary Note examines the sensitivity of the results to changes in cost weight parameters (Table 3). All parameters were scaled from 0.001 times to 1,000 times in each case.

## Supplementary Video

The file replays the simulation in Cases 1–4.

# Supplementary Note: Sensitivity to cost weight parameters

Yuki Ueyama

*Department of Mechanical Engineering, National Defense Academy of Japan, Yokosuka, Kanagawa, Japan*

This material examines the sensitivity of the results to changes in the cost weight parameters (Table 3). All parameters were scaled from 1/1,000 times to 1,000 times in each case. Thus, the new cost weight parameters were set to

$$[w_p', w_v', w_f'] = r \cdot [w_p, w_v, w_f],$$

where *r* is the scaling parameter between 1/1,000 and 1,000.

The effects of the scaling of the cost weight parameters were minimal (Figures S1–S6). Although the scales at *r* = 1/1,000 showed small movement magnitudes and higher muscle activations, respectively (Figures S1 and S6), we found consistent behaviour between 1/100 and 100 times (Figures S2–S5).

The stabilisation control was more sensitive to the cost weight parameters than was the default setting, which adopted only the terminal cost (Figures S7–S12). In low scales at *r* = 1/1,000 and *r* = 1/100, the stabilisation control of the position cost could not reproduce the bell-shaped velocity profiles, and the stabilisation control of the position and velocity costs changed the muscle activation pattern to a biphasic pattern from the triphasic pattern (Figures S7 and S8). At high scale (*r* = 1000), we failed to compute hand trajectory due to the lack of precision of the algorithm, and the muscle activation patterns were blunt (Figures S12).

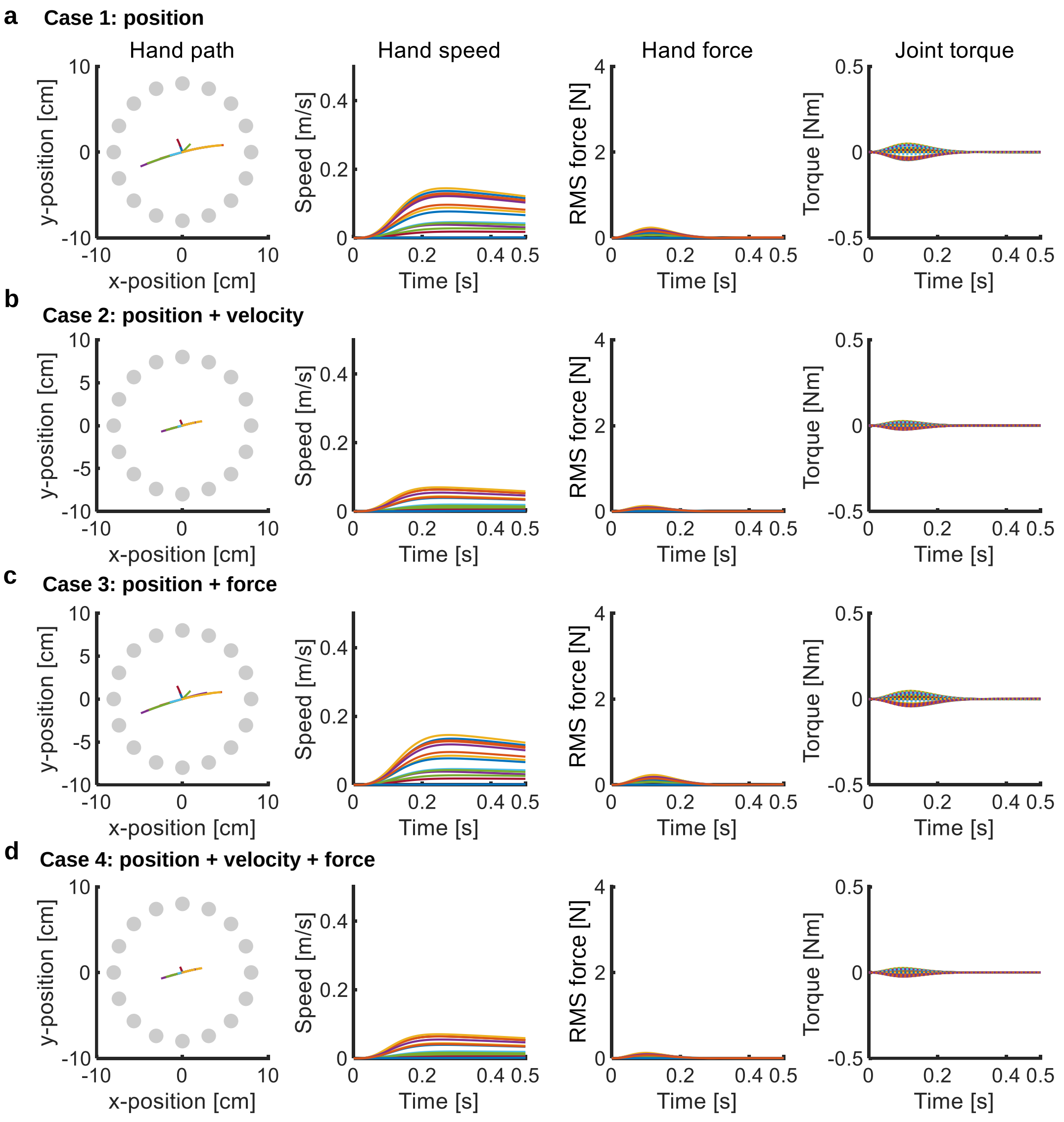


**Figure S1-A. Model behaviour at *r* = 1/1,000.** The format is identical to Figure 2 in main article.

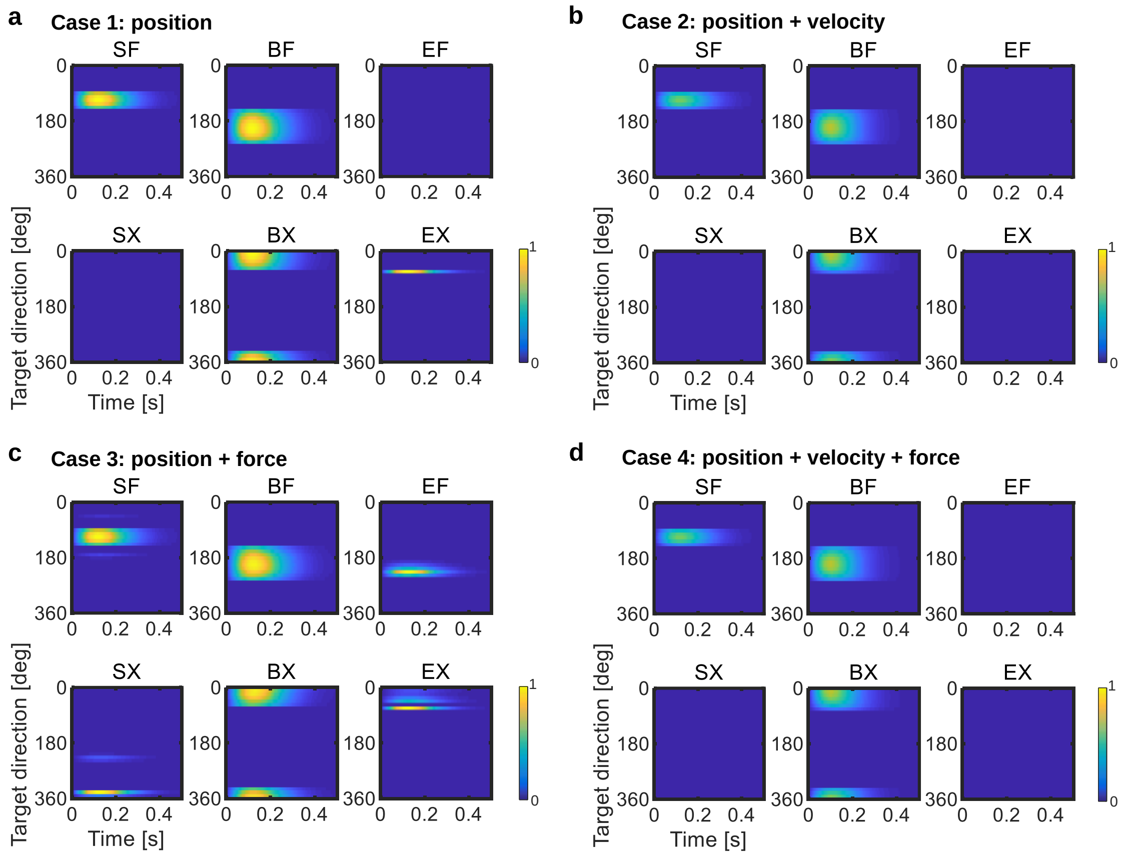


**Figure S1-B. Muscle activation patterns at $r$ = 1/1,000.** The format is identical to Figure 3 in main article.

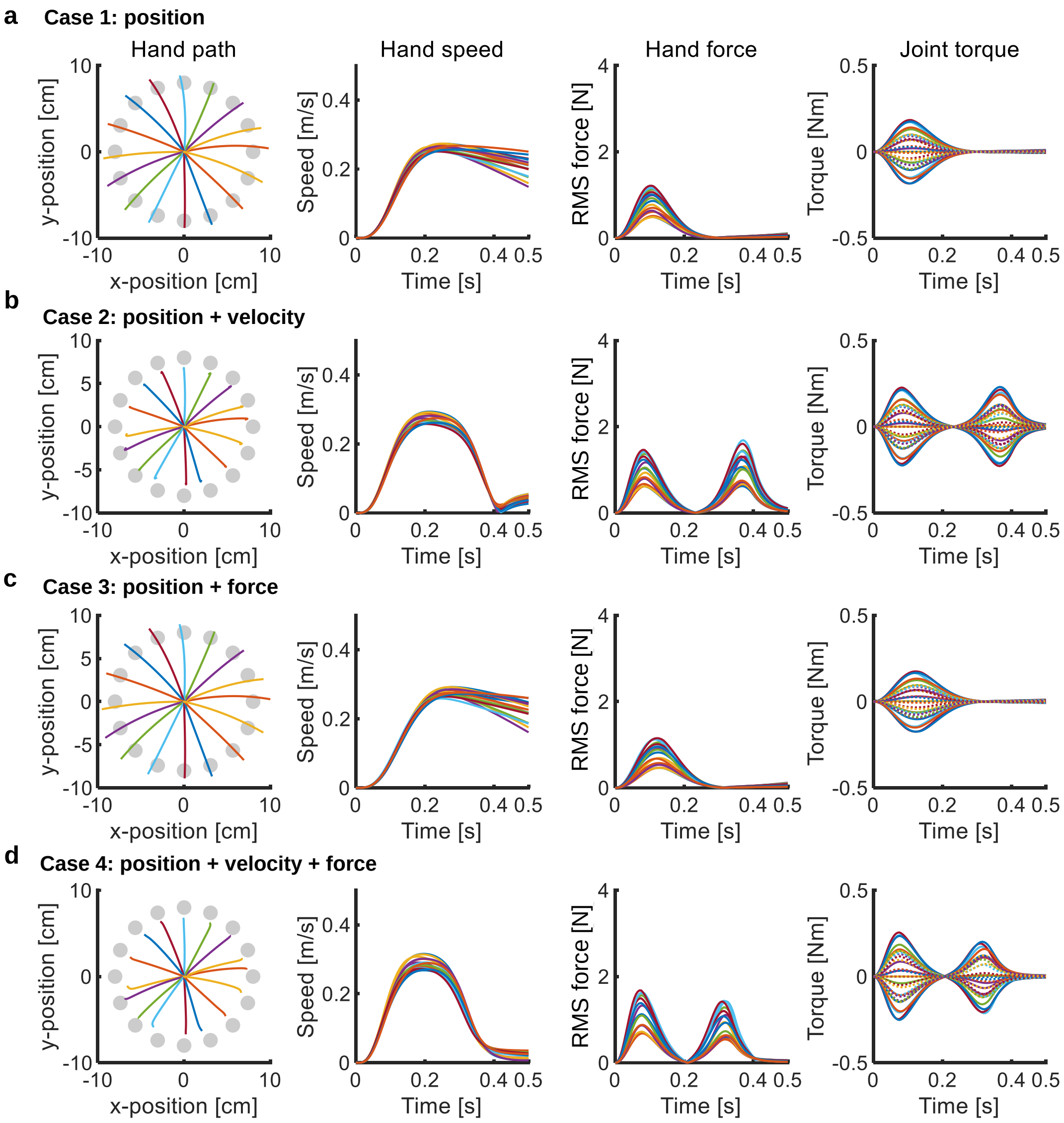


**Figure S2-A. Model behaviour at *r* = 1/100.** The format is identical to Figure 2 in main article.

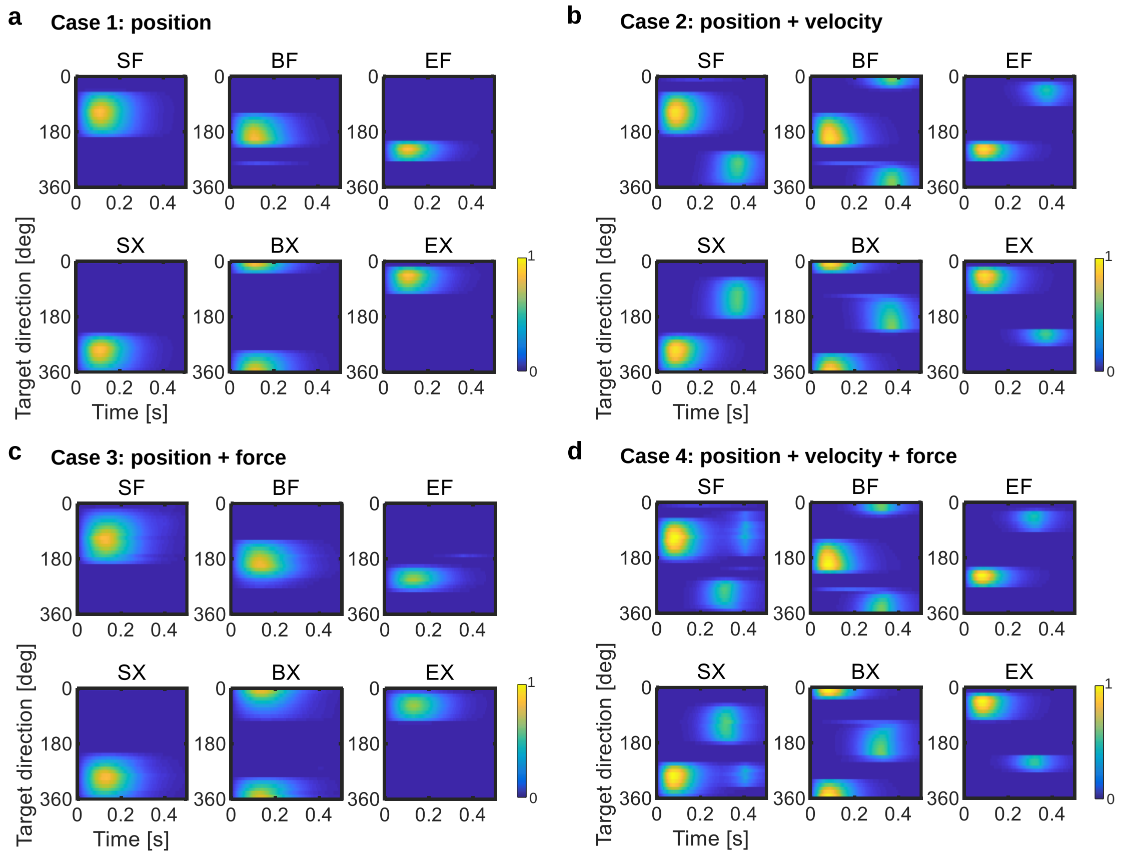


**Figure S2-B. Muscle activation patterns at *r* = 1/100.** The format is identical to Figure 3 in main article.

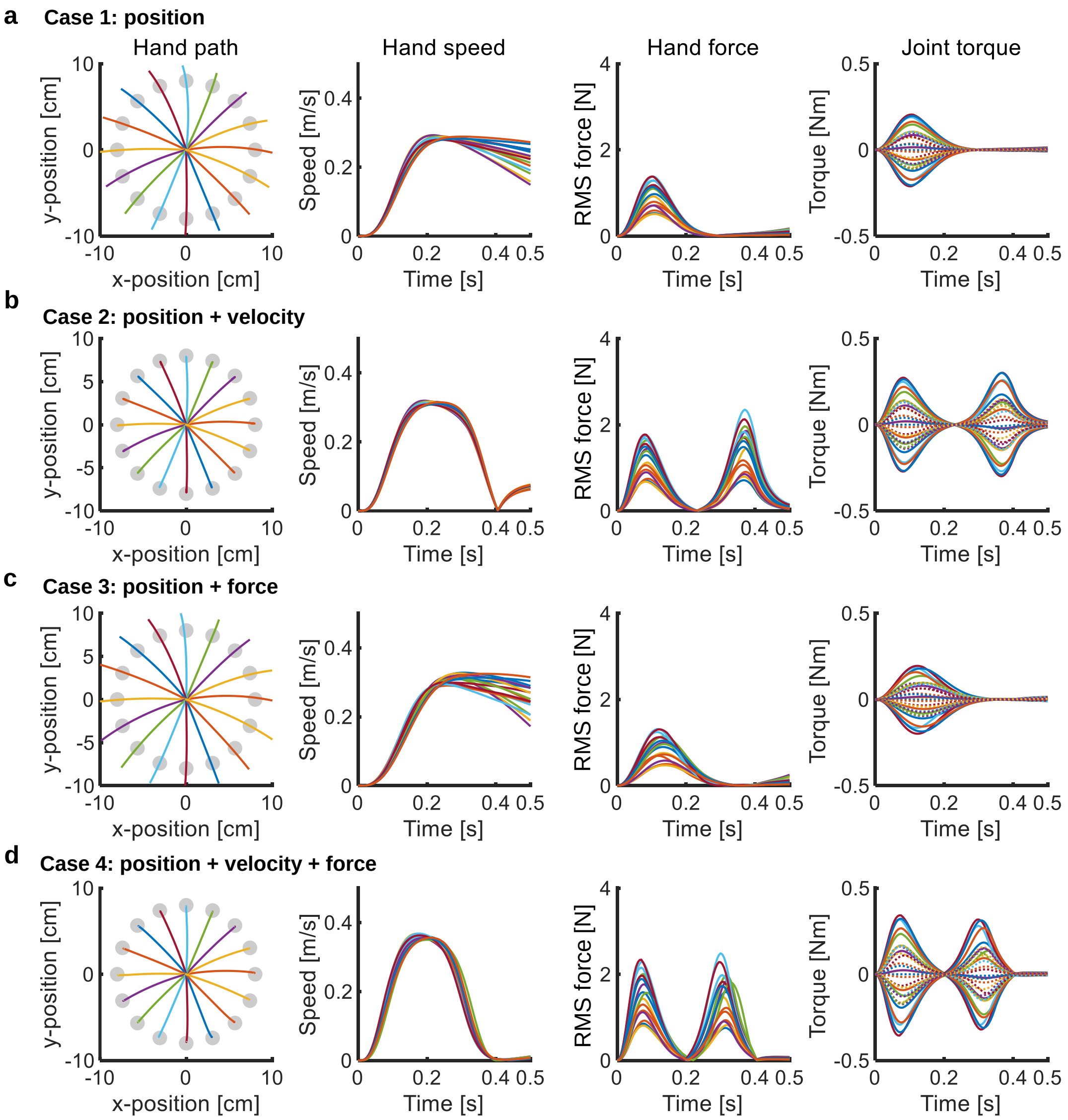


**Figure S3-A. Model behaviour at *r* = 1/10.** The format is identical to Figure 2 in main article.

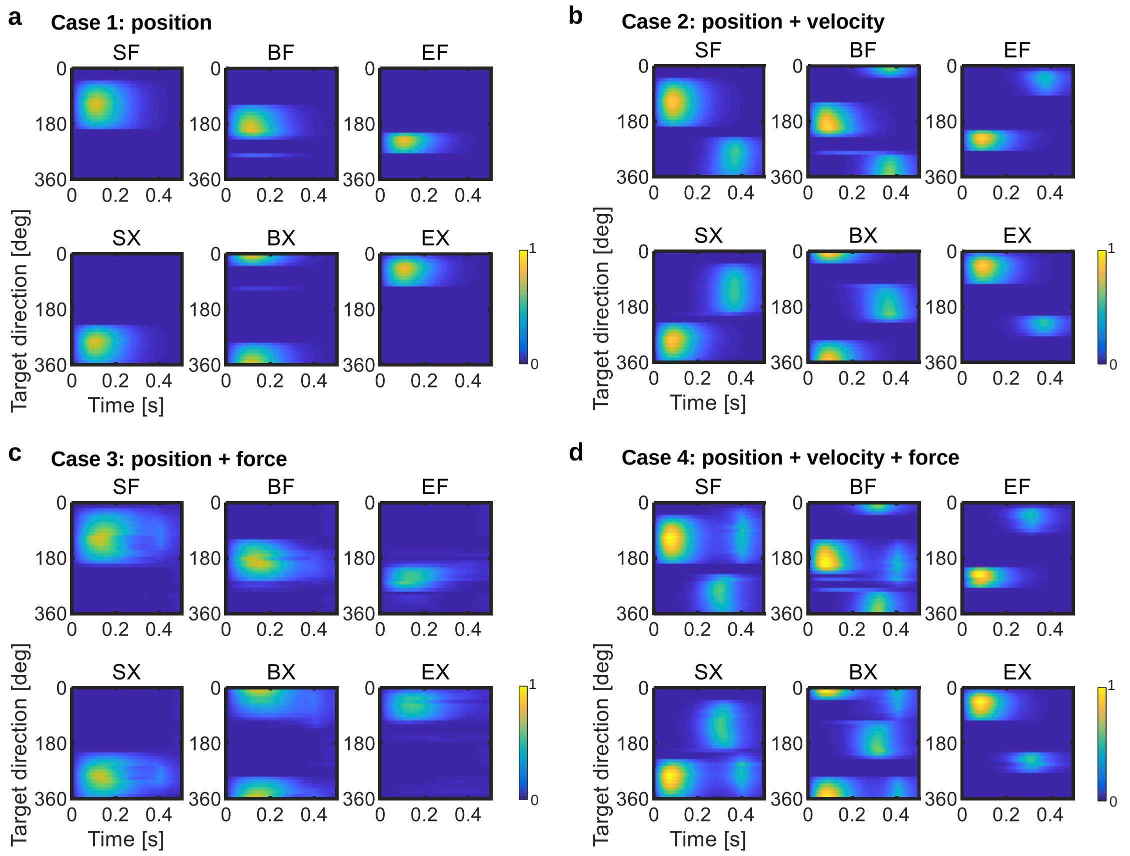


**Figure S3-B. Muscle activation patterns at *r* = 1/10.** The format is identical to Figure 3 in main article.

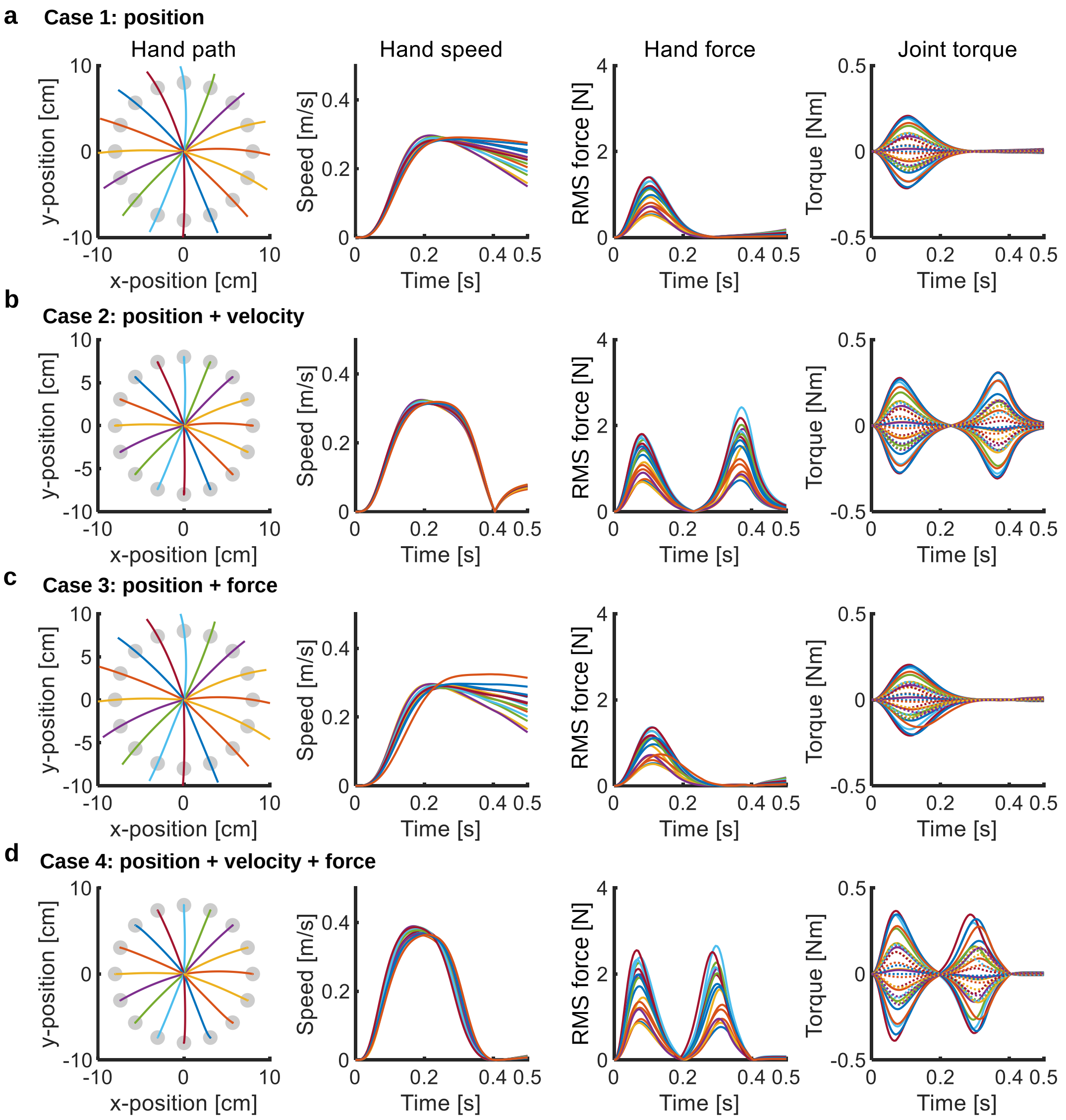


**Figure S4-A. Model behaviour at *r* = 10.** The format is identical to Figure 2 in main article.

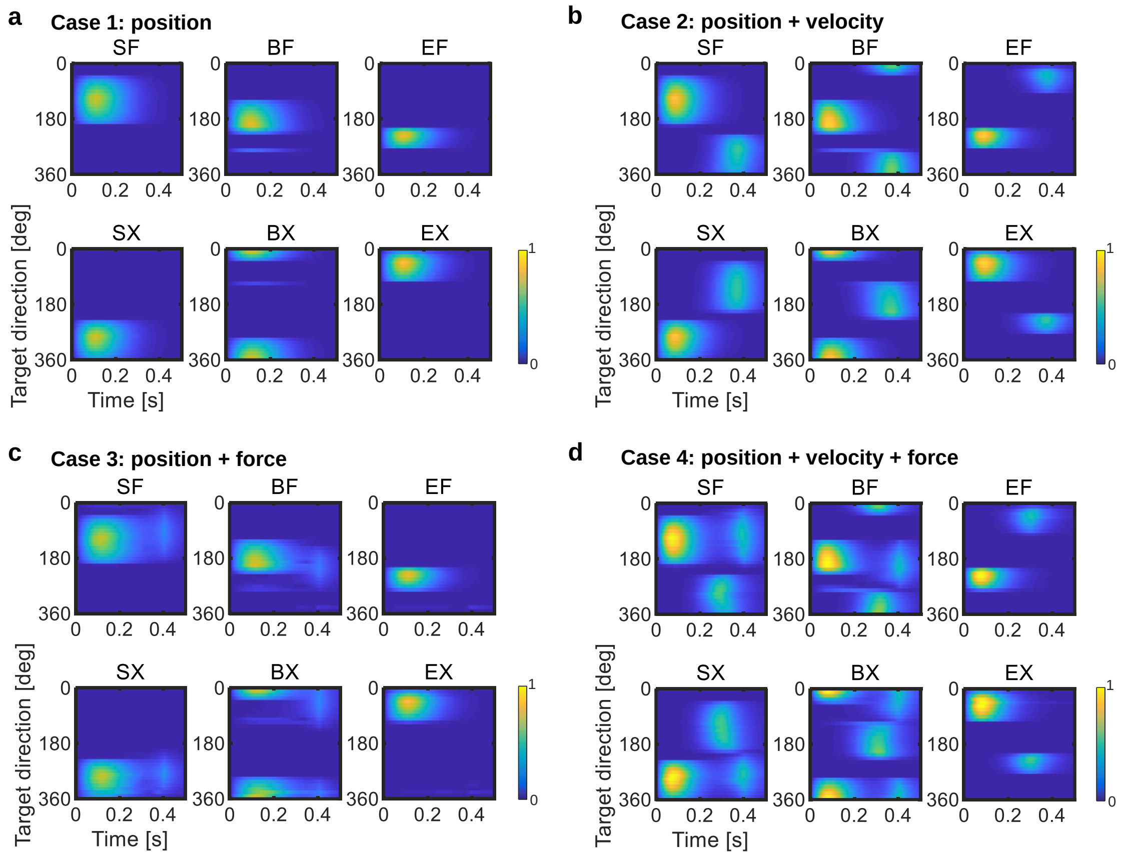


**Figure S4-B. Muscle activation patterns at *r* = 10.** The format is identical to Figure 3 in main article.

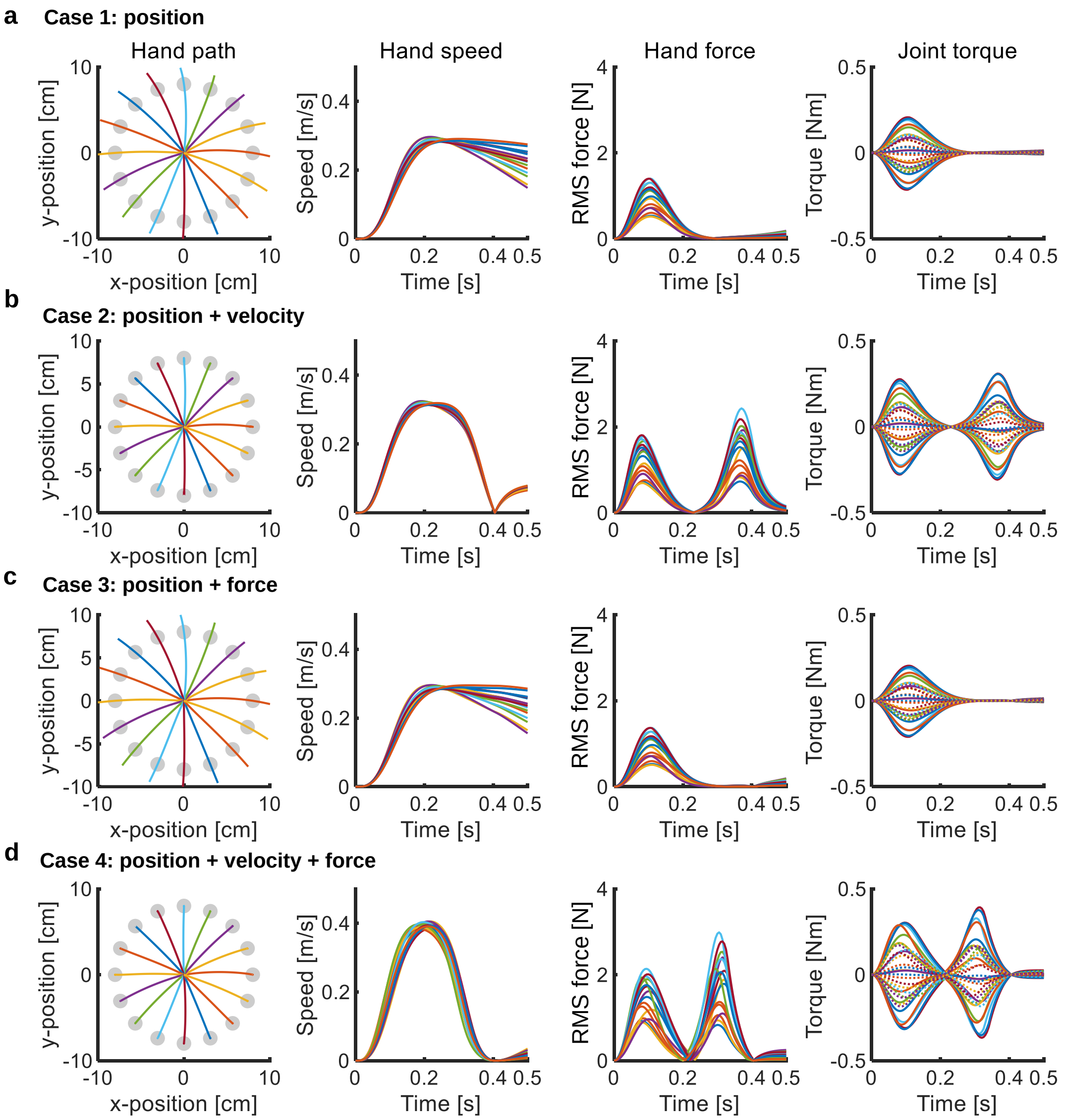


**Figure S5-A. Model behaviour at *r* = 100.** The format is identical to Figure 2 in main article.

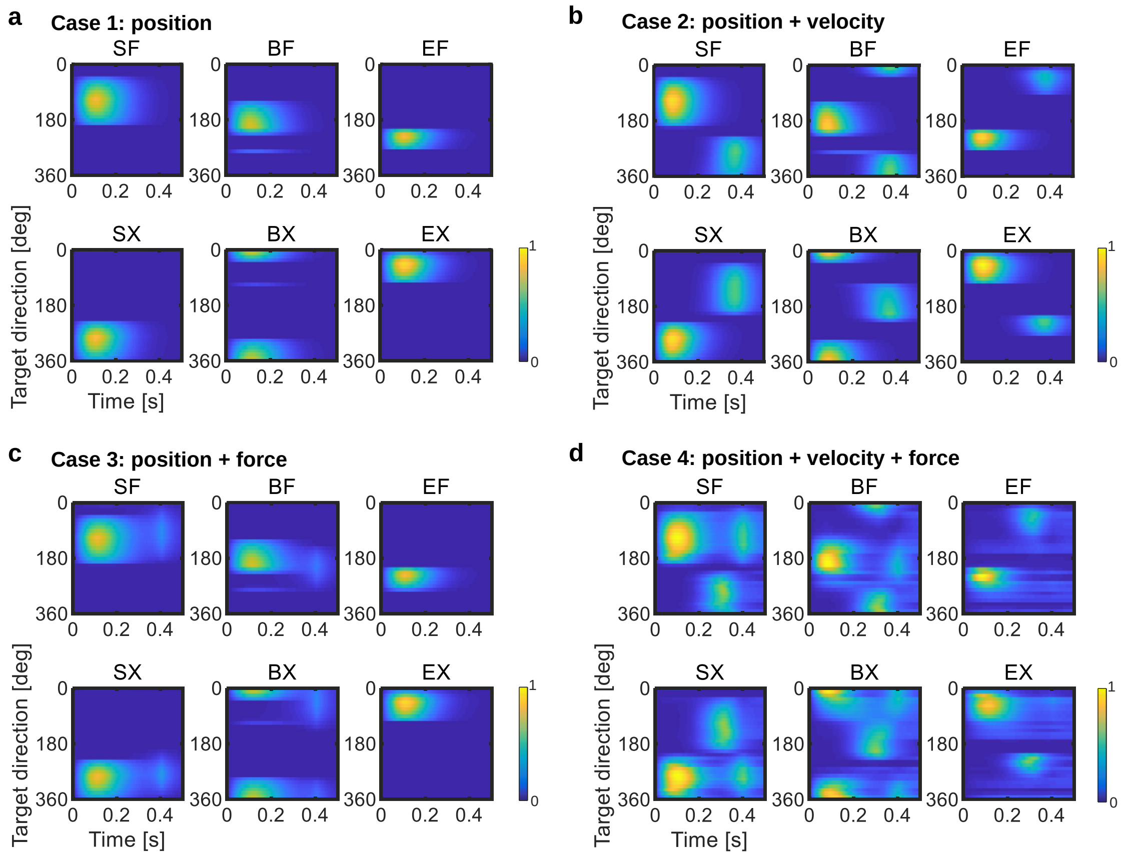


**Figure S5-B. Muscle activation patterns at *r* = 100.** The format is identical to Figure 3 in main article.

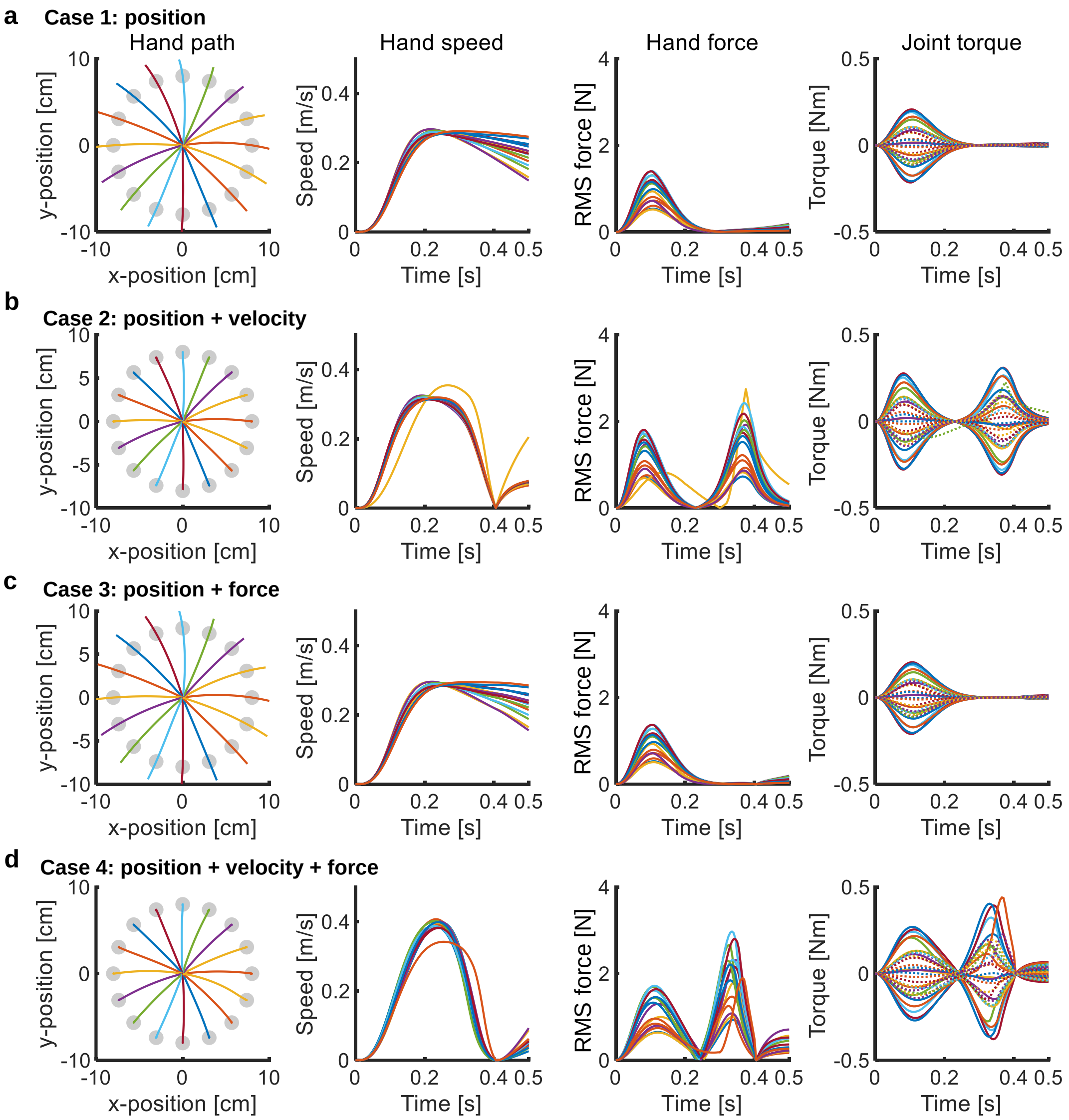


**Figure S6-A. Model behaviour at *r* = 1,000.** The format is identical to Figure 2 in main article.

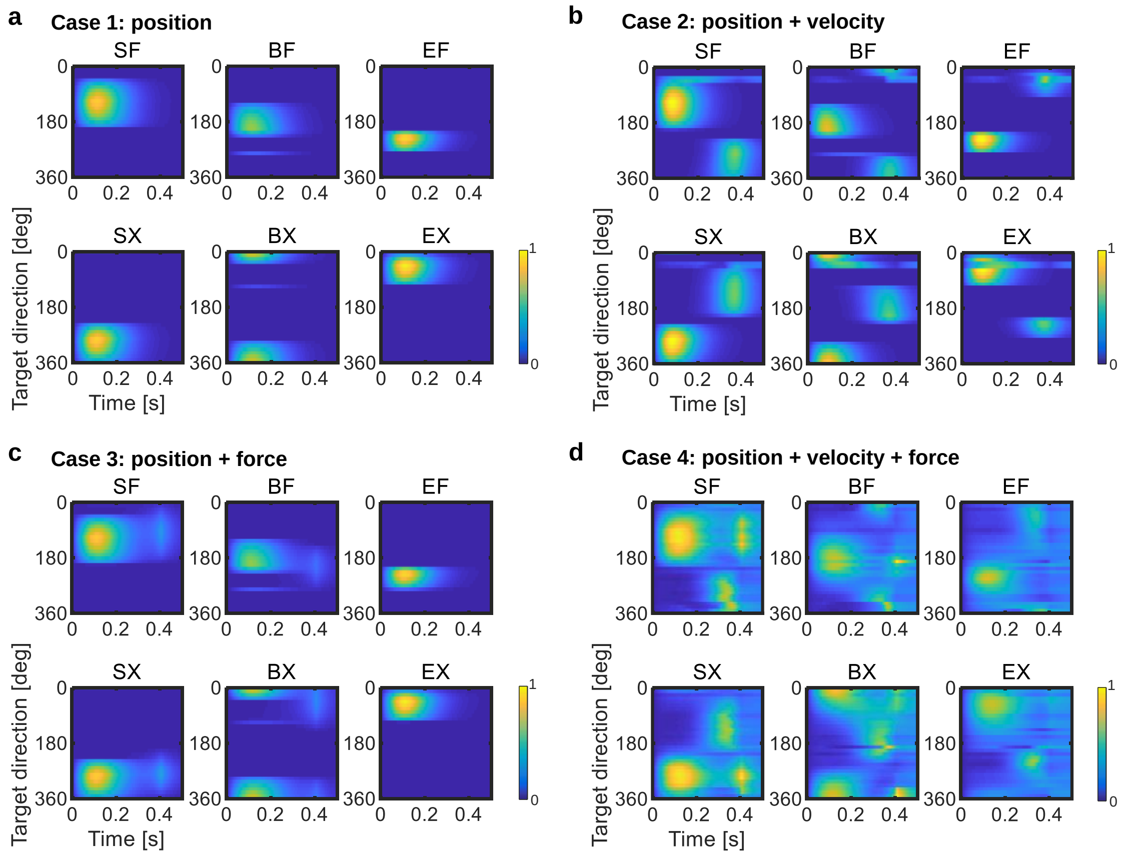


**Figure S6-B. Muscle activation patterns at *r* = 1,000.** The format is identical to Figure 3 in main article.

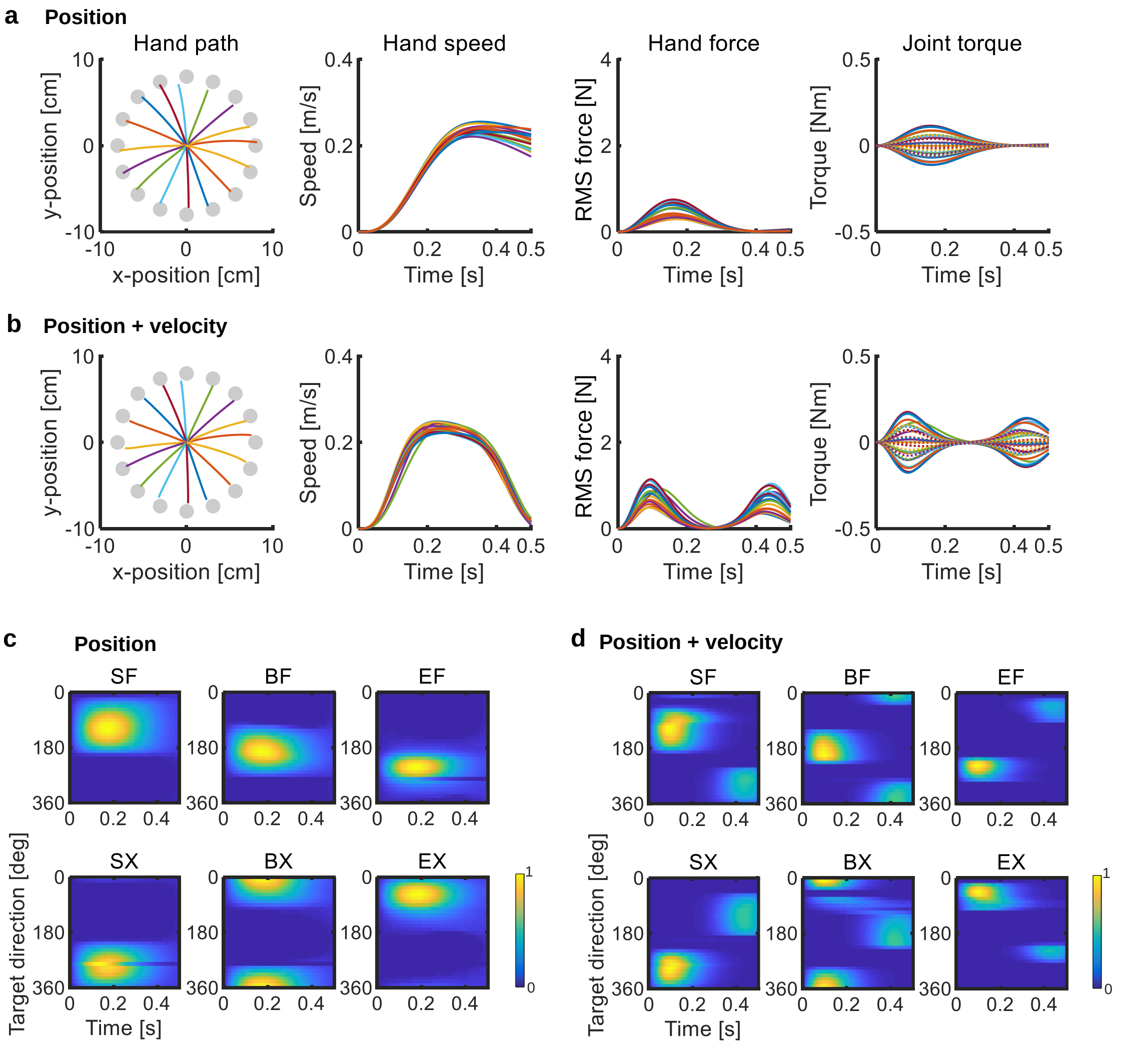


**Figure S7. Stabilisation control at *r* = 1/1,000.** The format is same as in Figure 5 in the main article.

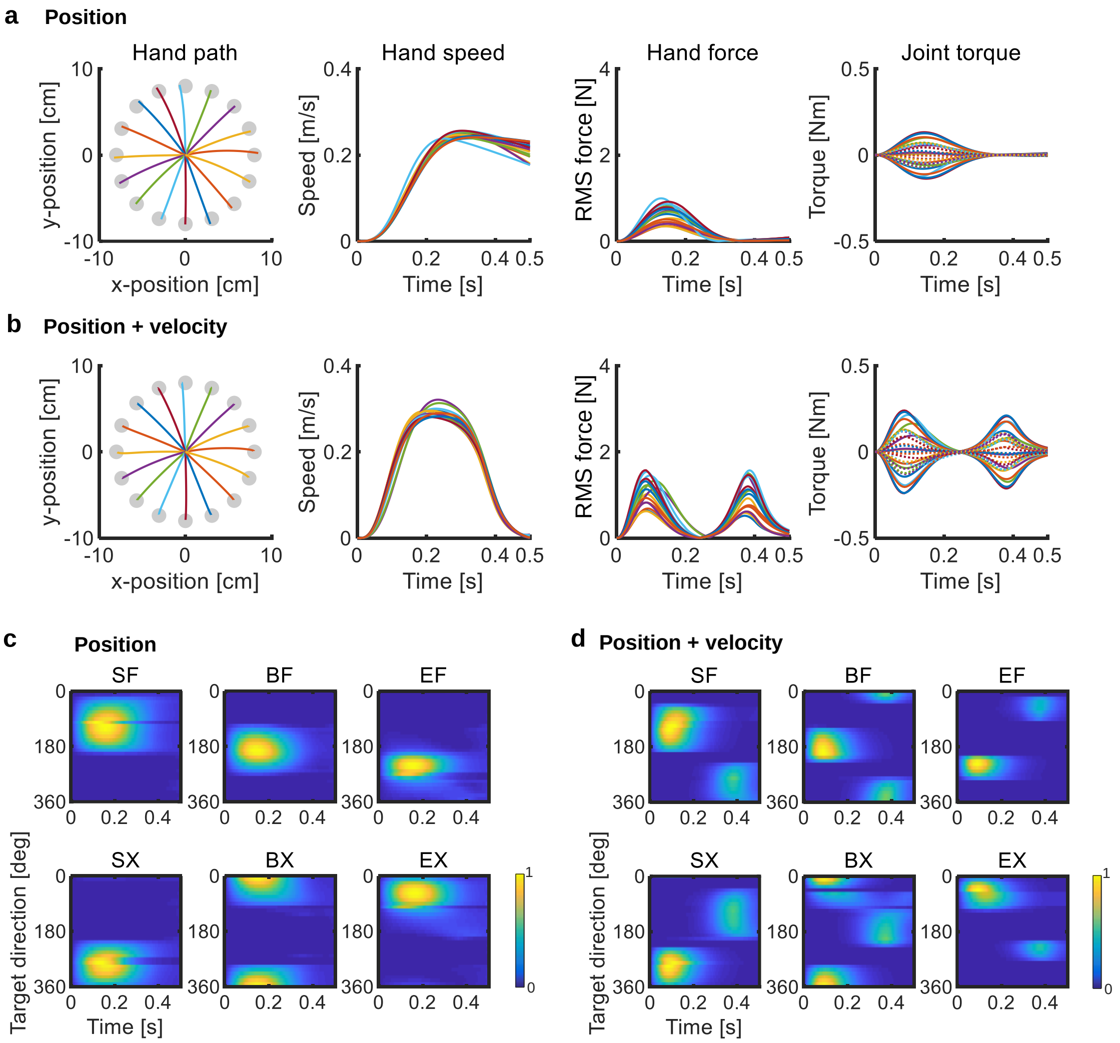


**Figure S8. Stabilisation control at *r* = 1/100.** The format is same as in Figure 5 in the main article.

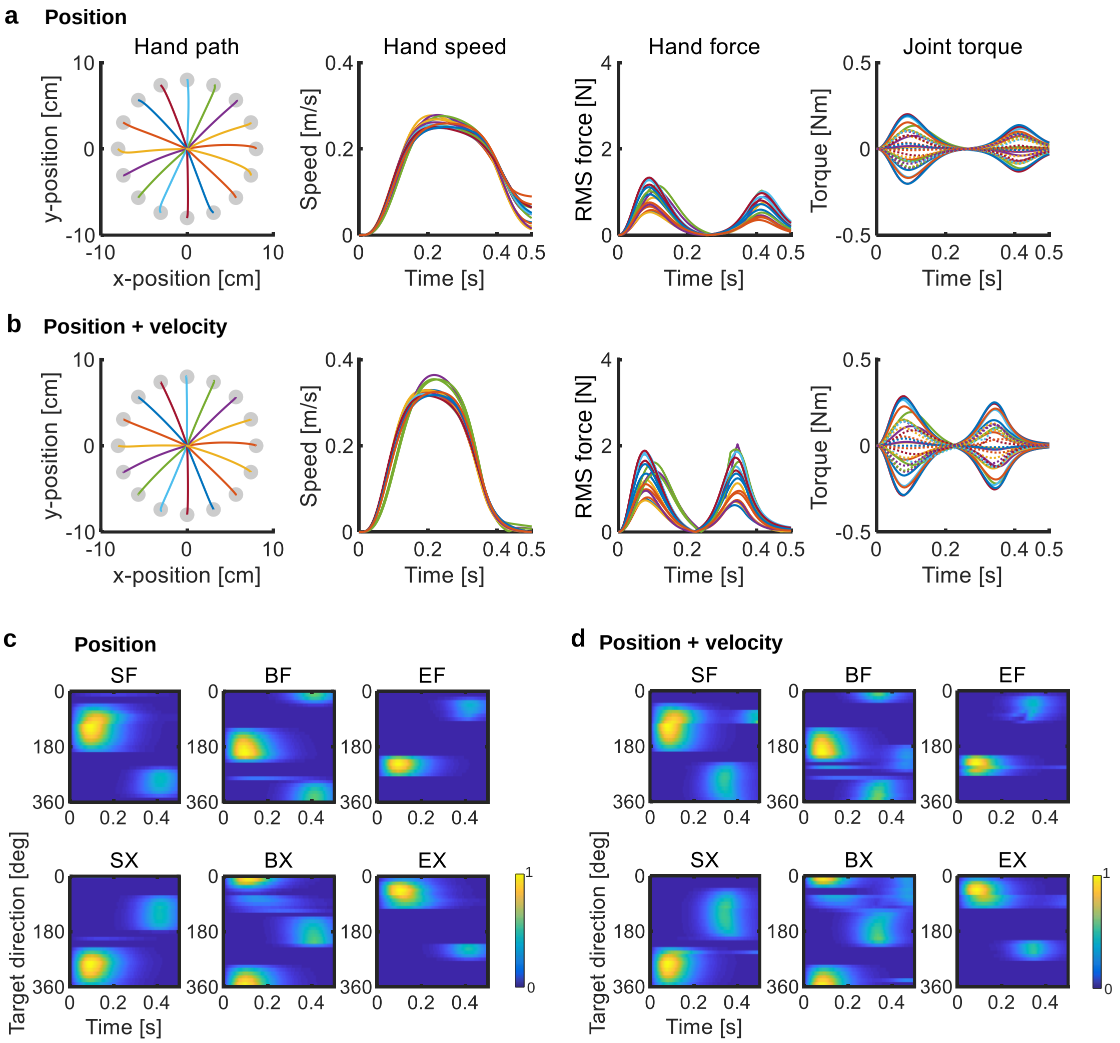


**Figure S9. Stabilisation control at *r* = 1/10.** The format is same as in Figure 5 in the main article.

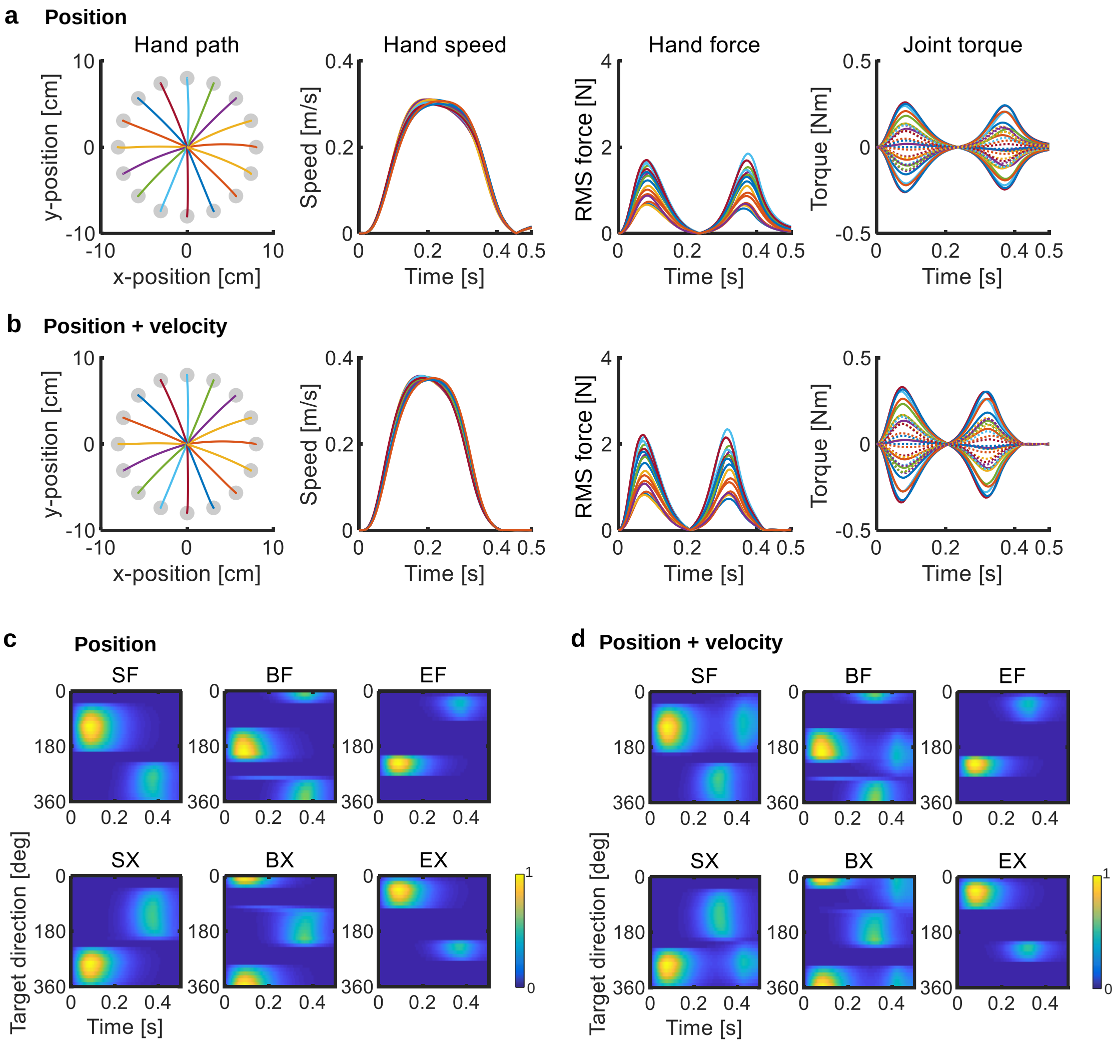


**Figure S10. Stabilisation control at *r* = 10.** The format is same as in Figure 5 in the main article.

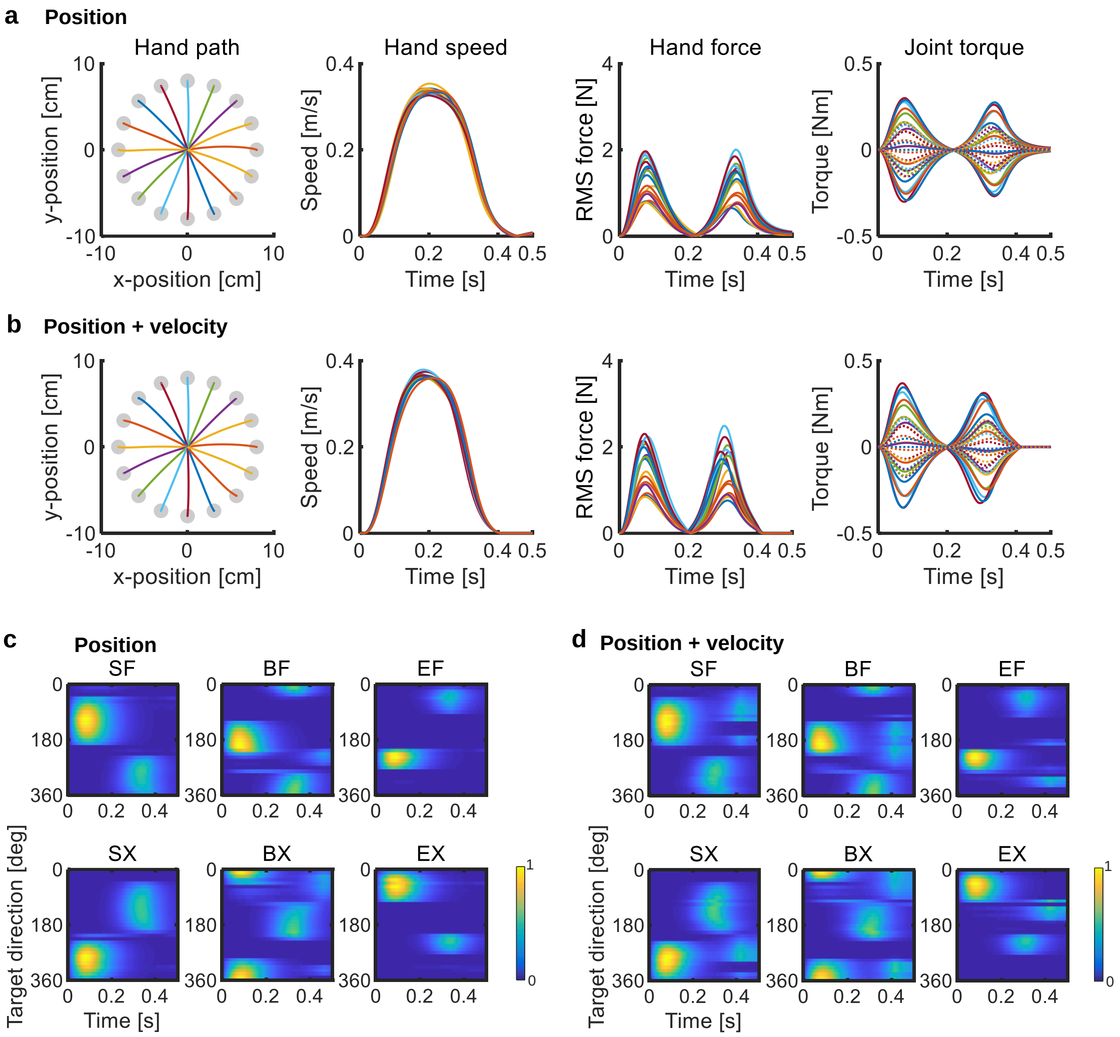


**Figure S11. Stabilisation control at *r* = 100.** The format is same as in Figure 5 in the main article.

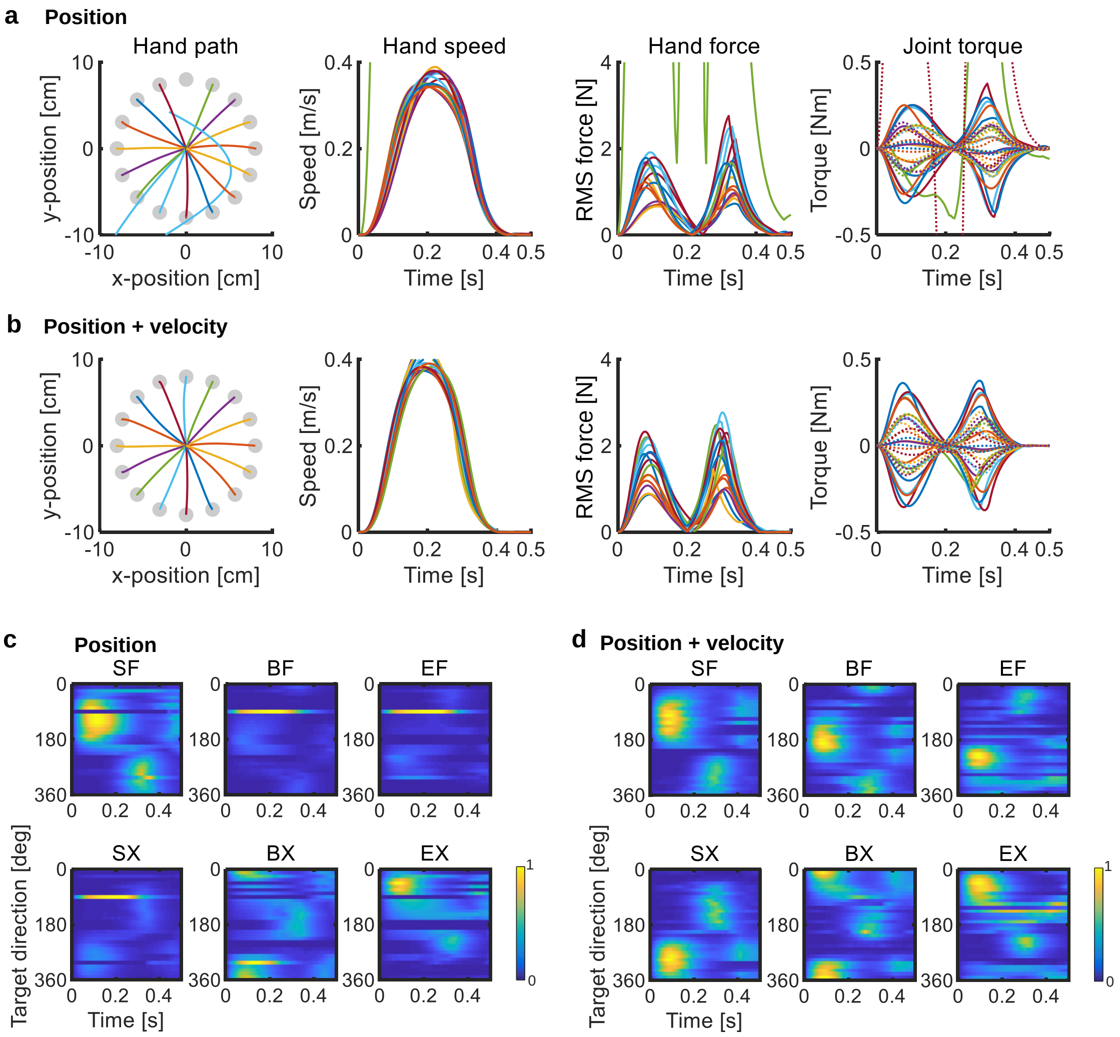


**Figure S12. Stabilisation control at *r* = 1,000.** The format is same as in Figure 5 in the main article.
